# The dual antifungal and antibiofilm activities of β-carotene against multidrug-resistant *Candida albicans* induce wound healing in a diabetic rat model: an in vitro and in vivo study

**DOI:** 10.1186/s12866-025-04447-w

**Published:** 2025-11-15

**Authors:** Khaled B. Al-Monofy, Ahmed A. Abdelaziz, Amal M. Abo-Kamar, Lamiaa A. Al-Madboly, Mahmoud H. Farghali

**Affiliations:** Department of Microbiology and Immunology, Faculty of Pharmacy, Tanta University, Tanta, Egypt

**Keywords:** Diabetic wounds, *Candida albicans*, Antimicrobial resistance, β-Carotene, Antifungal, Antibiofilm

## Abstract

**Background:**

Diabetes mellitus renders patients susceptible to chronic wounds and fungal infections, such as *Candida albicans* infections. The treatment of *C. albicans*-infected diabetic wounds is often problematic due to drug resistance and biofilm formation. Therefore, we studied the impact of β-carotene on treating diabetic wounds infected with strong biofilm-forming multidrug-resistant (MDR) *C. albicans*.

**Results:**

Our data showed that β-carotene (200 µg/ml) exhibited potent antifungal activity against the planktonic *C. albicans* cells, including MDR cells, with inhibition zones of 14 to 34 mm, which was emphasized by methylene blue staining and growth kinetics analysis. Using polystyrene plates and silicon catheter models, β-carotene showed a promising antibiofilm action by disrupting the established biofilms of *C. albicans* by 95% and 75.9%, respectively, which was confirmed by crystal violet staining, dual staining, and fluorescence staining approaches. The antibiofilm pathway of β-carotene was also examined, and it was found that β-carotene targeted the first stage of biofilm via reducing the adherence up to 90%, which was linked to downregulation of expression of the hypha-specific gene, *ALS3*, and blocking action of agglutinin-like protein 3 (Als3) via complexation (-9.8 kcal/mol). In addition, β-carotene disrupted the initiation step by inhibiting the yeast-to-hyphae transition and lowering the viability of sessile cells up to 80%. Furthermore, the maturation step of biofilm was embattled by β-carotene through a 95.6% reduction in the production of polysaccharide matrix. The in vivo model demonstrated the curative role of β-carotene in the healing process, which displayed a wound healing ratio of 89%, a lower fungal burden, and better histological features.

**Conclusion:**

Our findings demonstrated the therapeutic potential of β-carotene for treating diabetic wounds infected with strong biofilm-forming MDR *C. albicans*.

**Supplementary Information:**

The online version contains supplementary material available at 10.1186/s12866-025-04447-w.

## Introduction

A metabolic condition known as diabetes mellitus (DM) is caused by a buildup of high glucose levels in tissues and organs. A major morbidity issue that is becoming more prevalent globally is delayed wound healing in DM patients [1]. Diabetic patients are also more susceptible to fungal infections, as their immunosuppressive condition and high blood sugar levels affect the balance between host and yeast, allowing the commensal *Candida albicans* pathogen to transition and cause infection [2]. Furthermore, the emergence of resistance to *C. albicans* treatment in diabetic patients raises concerns that may significantly impact morbidity and mortality rates [3].

Drug resistance mechanisms have been developed by *C. albicans*, which employs a variety of tactics to counteract the effects of different drug classes [4]. Although numerous treatment approaches, such as topical therapies, regenerative medicine, and hyperbaric oxygen, have been documented [5], no therapeutic method has proven successful in treating persistent wounds and infections in patients with DM [6]. Therefore, it is essential to comprehend the mechanisms of resistance in *C. albicans* in order to create effective therapeutic strategies.

Among the various causes of multidrug resistance in *C. albicans* are biofilms that impede the antifungal’s ability to reach the target [7]. Fungal biofilm production is essential to pathogenesis, and the majority of *C. albicans*-caused illnesses are linked to the creation of biofilms [8]. The formation of biofilm by *C. albicans* passes through four main steps. The initial step is the adherence step, at which the yeast cell adheres to a substrate to form a basal layer of fungal cells. The second step involves the proliferation of fungal cells and the formation of hyphae (initiation step). During the third step (maturation step), the polysaccharide matrix is accumulated, and the biofilm becomes mature. The final step involves the release of non-adherent yeast cells from the biofilm into the environment, where they can colonize additional surfaces (dispersal step) [9]. Compared to their planktonic counterparts, fungal biofilms exhibit decreased susceptibility to existing antifungal medications [10]. Complex and multifaceted mechanisms, including a slower growth rate, increased cell density, a variety of stress responses, persister cell presence, extracellular matrix secretion, upregulation of the membrane transporter system and efflux pumps, and differential regulation of drug targets, are responsible for this fungal biofilm’s resistance to antifungal medications [11].

Although new antifungal treatments are constantly being developed, the majority of antifungals on the market are either ineffective against *C. albicans* biofilms because they require large concentrations to be effective [12] or have serious side effects because of their toxicity [13]. Furthermore, the eukaryotic nature of fungal cells limits the number of therapeutic targets that may be investigated to specifically destroy the pathogen, making it difficult to find effective antifungals because they evolve closely with their human hosts [14]. Therefore, the development of novel antifungals with minimal toxicity and great therapeutic effectiveness against *C. albicans* biofilm is urgently needed.

β-Carotene is a prominent member of the carotenoid family with a molecular weight of 536.88 and chemical formula C_40_H_56_ [15]. The antimicrobial properties of β-carotene have been documented in numerous studies against various microbial species, such as *Staphylococcus aureus*, *Streptococcus agalactiae*, *Pseudomonas aeruginosa*, *Klebsiella pneumoniae*, *Fusarium oxysporum*, *Aspergillus niger*,* and Penicillium chrysogenum* [16, 17]. β-Carotene’s antimicrobial effect is linked to its capacity to dissolve in the lipids of the plasma membrane, increasing penetration through the membrane that leads to inhibiting or destroying the growth of some pathogenic microorganisms [18]. Therefore, the goal of the current study was to examine the potential anticandidal and antibiofilm actions of β-carotene against MDR *C. albicans* colonizing the diabetic wounds in a rat model.

## Materials and methods

### Microorganism

Yeast isolates, isolated from wounds, were obtained from the microbiological laboratory at Tanta University, Egypt, and were cultured on Sabouraud dextrose agar (SDA). The identification of *C. albicans* isolates was performed using CHROMagar™ Candida medium, Gram-staining, germ tube test, and matrix-assisted laser desorption/ionization time-of-flight mass spectrometry (MALDI-TOF MS). For the preparation of inoculum, *C. albicans* was cultured on SDA, incubated at 37 °C for 48 h before each experiment, and was standardized using 0.5 McFarland with a final concentration of approximately 10^6^ colony-forming units per milliliter (CFU/ml) [19–21].

### Antifungal Susceptibility Testing (AFST) for C. albicans and detection of MDR isolates

The AFST was performed on Mueller-Hinton agar supplemented with 2% glucose (2%) and methylene blue (0.00005%) using the Kirby-Bauer disk diffusion method according to CLSI (2009). The examined antifungal agents were miconazole (MIC 30 µg), amphotericin-B (AMB 100 µg), nystatin (NS 50 IU), fluconazole (FLC 10 µg), and itraconazole (IT 10 µg) [7]. After that, the MDR isolates were identified according to Arendrup & Patterson (2017), who stated that the *Candida* isolates are defined as MDR if they are nonsusceptible to 1 or more antifungal agents in 2 or more antifungal classes [22]. *Candida albicans* (MTCC 227) was used as a standard strain.

### Antifungal activity of β-carotene against C. albicans using agar well diffusion assay

The antifungal activity was performed by agar well diffusion method against the isolated *C. albicans*, as El Zawawy et al. prescribed [23]. Firstly, the SDA with 8-mm wells was inoculated with 100 µl of each *C. albicans* strain (0.5 McFarland). Then, a volume of 100 µl of β-carotene (200 µg/ml), purchased from Sigma-Aldrich and prepared by dissolving the powder in 10% DMSO, was added to one well, and the other well was filled with 10% DMSO as a negative control. After 24 h of incubation at 37 °C, the results were detected, and the diameter of the inhibition zone around each well was measured. (Amphotericin-B was used as a positive control).

### Minimum Inhibitory Concentration (MIC) determination via broth microdilution assay

The MIC of β-carotene against *C. albicans* isolates was tested by broth microdilution assay, as detailed by Sun et al. [24]. In a 96-well microtiter, a serial dilution of β-carotene was prepared from a stock (20 mg/ml), starting from 100 µg/ml and ending with 3.125 µg/ml using Sabouraud dextrose broth (SDB). The concentration of prepared *C. albicans* suspension was adjusted to 4 × 10^3^ CFU/ml by SDB, and 100 µl of *C. albicans* suspension was placed into wells filled with 100 µl of SDB, which contained β-carotene, and fungal growth or inhibition was observed after incubation at 37 °C for 24 h. At the first well in each column, *C. albicans* suspension in SDB (200 µl) acted as a growth control, and un-inoculated SDB (200 µl) was added to the last well as a negative control. Finally, the MIC was determined as the lowest concentration at which no visual growth was observed, and the experiments were repeated in triplicate. Amphotericin-B was used as a positive control.

###  Analysis of growth kinetics using time-kill assay against MDR isolates

The anticandidal activity was assessed with 0, 0.25, 0.5, 1, and 2 MICs of β-carotene against MDR *C. albicans* (CA 2, 3, 5, 6, 8, 14, 17, and 20) and *C. albicans* MTCC 227, and the resultant growth profile for 12 h was plotted as detailed by Rajeh et al. [25]. β-Carotene was prepared in desired concentrations in a vial containing 15 ml SDB, and then the cell suspension was adjusted to an optical density (OD) of 0.15 at 600 nm, and 10% DMSO-containing medium acted as a control. Then, the vials were incubated at 37 °C for 12 h, and a volume of 1 ml was taken from each vial to measure OD at 600 nm every 2 h. Finally, the growth profile was plotted using Microsoft Excel. (The experiments were repeated in triplicate).

### Biofilm formation ability of C. albicans using the Crystal Violet (CV) technique

According to Anggraini et al. the CV technique was used to assess the ability of *C. albicans* isolates to generate biofilms [26]. A volume of 200 µl of the prepared yeast suspension (10^6^ cells/ml) in SDB, supplemented with 1% glucose, was inoculated in a flat-bottom 96-well microplate, and then the plate was incubated at 37 °C under static conditions to allow biofilm formation. Following a 24-h incubation period, the extra media was discarded, and the wells were air-dried after being cleaned three times with 200 µl of phosphate-buffered saline (PBS) to get rid of any detached cells using a micropipette. Following that, 150 µl of methanol was added for 20 min to enhance biofilm attachment, and the plate was left to air dry. Then 180 µl of 0.1% CV was applied to each well for 15 min. After discarding the CV, the residual stain was eliminated with three rounds of washing with sterile distilled water (SDW). Finally, a microtiter reader (SunriseTM, TECAN, Switzerland) was used to measure the OD at 595 nm after adding 180 µl of 33% (vol/vol) glacial acetic acid to dissolve the embedded dye within biofilms. Uninoculated SDB were used as negative controls, and each strain of *C. albicans* filled up three wells. *C. albicans* (MTCC 227) was used as a standard strain. The resulting biofilms were classified according to the cut-off value (OD value of the negative control (ODC)). (The experiments were repeated in triplicate).

**Table:** 

Biofilm is negative when OD ≤ ODC.
Biofilm is slightly positive when ODC ≤ OD ≤ 2 x ODC.
Biofilm is moderately positive when 2 × ODC ≤ OD ≤ 4 × ODC.
Biofilm is strongly positive when 4 × ODC ≤ OD.

### Antibiofilm activity of β-carotene against strong biofilm-forming C. albicans using microtiter plate and silicon catheters assays

The effect of β-carotene on the mature biofilms of *C. albicans* (CA 2, 3, 5, 6, 8, 14, 17, 20, and MTCC 227) was evaluated, as prescribed by Feldman et al. [27]. At first, the biofilm was established as mentioned above, and the wells were washed twice with PBS, and then a volume of 200 µl of fresh SDB containing β-carotene (100 and 200 µg/ml) was added. The SDB containing 10% DMSO served as a control. After the plate was incubated for 24 h at 37 °C, the biofilms were measured using the 1% CV assay as stated above, and the assay was performed in triplicate by measuring OD at 595 nm. The percentage of eradication in the biofilm was calculated by using the following formula;

$$\begin{aligned} &\mathrm{Percentage}\;\mathrm{of}\;\mathrm{biofilm}\;\mathrm{eradication}\\&=\;\frac{\mathrm{OD}\left(\mathrm{untreated}\right)\;-\;\mathrm{OD}\left(\mathrm{treated}\right)}{\mathrm{OD}\;\left(\mathrm{untreated}\right)}\;\times100\end{aligned}$$

$$\begin{aligned} \mathrm{OD}\;(\mathrm{untreated})\;=\;&\mathrm{OD}\;\mathrm{at}\;595\;\mathrm{nm}\;\mathrm{of}\;\\&\mathrm{untreated}\mathit\;C\mathit.\mathit\;albicans.\end{aligned}$$

$$\begin{aligned} \mathrm{OD}\;\left(\mathrm{treated}\right)\;=\;&\mathrm{OD}\;\mathrm{at}\;595\;\mathrm{nm}\;\mathrm{of}\;\mathrm\beta-\mathrm{carotene}\\&-\mathrm{treated}\;\mathrm{or}\;10\%\;\mathrm{DMSO}\\&-\mathrm{treated}\;C\mathit.\mathit\;albicans.\end{aligned}$$

Furthermore, the antibiofilm ability of β-carotene was emphasized using silicon catheters, as described by Wongchai et al. with a few modifications [28]. Silicon catheter pieces with a 50 mm incision were immersed in a 6-well microplate containing 5 ml of yeast culture in SDB supplemented with 1% glucose (1 × 10^6^ cells/ml). After that, the plate was incubated under static conditions at 37 °C to enable the formation of biofilms on silicon catheters. The wells were washed twice with PBS, and a volume of 5 ml of fresh SDB containing either β-carotene (100 and 200 µg/ml) or 10% DMSO (control) was added. Following a 24-h incubation period at 37 °C, the wells were rinsed with 0.85% NaCl, the catheter segments were stained for 15 min using a 0.1% CV, rinsed with 0.85% NaCl, and resolved in 95% ethanol. The OD at 595 nm was then used to determine the biofilm eradication utilizing the aforementioned formula. The statistical analysis was performed by comparing the percentage of biofilm eradication after treatment of the *C. albicans* (untreated) biofilms with β-carotene (100 and 200 µg/ml) with the percentage of biofilm eradication after treatment the *C. albicans* biofilms with the negative control (10% DMSO). (Amphotericin-B was used as a positive control).

### Light Microscopy (LM) to evaluate the effect of β-carotene on biofilm, adherence, and hyphae formation of C. albicans 

As described by Venkatramanan et al. the effect of β-carotene on the biomass of *C. albicans* biofilms (CA 2, 3, 5, 6, 8, 14, 17, 20, and MTCC 227) employing CV staining was detected utilizing the light microscopic examination method [29]. In brief, a 6-well microtitre plate with glass coverslips was filled with 2 ml of the prepared *C. albicans* suspension in SDB + 1% glucose, and the plate was then incubated at 37 °C in a static state. Following the incubation period of 24 h at 37 °C, the wells were twice rinsed with PBS before being filled with 2 ml of fresh SDB, both with β-carotene (100 and 200 µg/ml) and with 10% DMSO as a control. After 24-h incubation at 37 °C, the media were removed from treated and untreated wells to remove planktonic cells, and the glass coverslips were rinsed with SDW and then stained with 0.1% CV. Lastly, after two rounds of washing of excessive CV with SDW and air drying, the adherent biofilms on glass coverslips were seen under a LM at 1000-x. Furthermore, the antibiofilm activity of β-carotene was confirmed via dual staining technique using Maneval’s stain and Congo red, per the instructions provided by Manhas et al. [30]. Following the establishment of the β-carotene-treated and 10% DMSO-treated *C. albicans* biofilms as previously mentioned, the biofilms were washed with SDW, and then the biofilms were fixed for 30 min at room temperature using formaldehyde solution (4%). After air-drying, the fixed biofilms were dyed with 1% Congo red stain and allowed to dry, and then stained with Maneval’s stain for 10 min. After that, the coverslips were cleaned by gentle washing with SDW and dried at room temperature. Finally, the biofilms were inspected under a LM at 1000-x magnification. In case of LM examination of the effect of β-carotene on adherence, a 6-well microtitre plate with glass coverslips was filled with 2 ml of the prepared *C. albicans* suspension in SDB + 1% glucose, both with β-carotene (0.25 and 0.5 MICs) and with 10% DMSO as a control, and the plate was incubated in a static condition at 37 °C. Then, the media was removed from the wells to eliminate the planktonic cells after a 24-h incubation period at 37 °C, and the glass coverslips were rinsed with SDW and stained with 0.1% CV. After the removal of excessive stain and air-drying, the adhering cells on glass coverslips were finally observed under a LM [31]. In case of hyphae detection, the impact of β-carotene on yeast-to-hyphal phase transition was investigated using LM, as detailed by Sun et al. [24]. An overnight cultured *C. albicans* was diluted (2 × 10^6^ CFU/ml) in RPMI-1640 medium + 15% fetal bovine serum (FBS), both with β-carotene (0.25 and 0.5 MICs) and 10% DMSO as a control. After 4 h of incubation at 37 °C with constant shaking at 160 rpm, the phase transition in treated and untreated cells was visualized using a LM at 1000-x magnification. (Amphotericin-B was used as a positive control).

### Confocal Laser Scanning Microscopy (CLSM) of biofilm cells

According to Sun et al. the CLSM evaluated the impact of β-carotene on the pre-established biofilms of *C. albicans* (CA 14) [32]. Briefly, the β-carotene (100 and 200 µg/ml)-treated and 10% DMSO-treated biofilms were established in an 8-well chamber slide, as mentioned above, where 10% DMSO acted as a negative control. Following the three times washing with PBS to remove non-adherent cells, the biofilm was co-stained for 15 min in the dark using 30 µl of each of propidium iodide (PI) and acridine orange (AO), and the treated and untreated biofilms were screened by CLSM (DMi8; Leica Microsystem). The biofilm was fully visualized in three dimensions using Z-stacks, and the fluorescence intensity was ascertained using ImageJ’s histogram. (Amphotericin-B was used as a positive control).

### Impact of β-carotene on the adherence ability of C. albicans on polystyrene plates

The effect of β-carotene on the adherence of *C. albicans* (CA 2, 3, 5, 6, 8, 14, 17, 20, and MTCC 227) was inspected via CV staining using 96-well and 6-well microtitre plates as prescribed by Awadelkareem et al. [31]. The ultimate concentration of 5 × 10^6^ cells/ml was achieved by adjusting an overnight *C. albicans* culture in SDB medium (supplemented with 1% glucose), containing either β-carotene (0.25 and 0.5 MICs) or 10% DMSO as a control. A 96-well polystyrene plate with a flat bottom was filled with 200 µl of *C. albicans* cell suspensions, and the plates were kept at 37 °C for 24 h. After that, the planktonic cells were discarded, and 200 µl of PBS was used to gently wash the wells. Following that, adherent cells were stained with 0.1% CV (200 µl), and further incubation was carried out for 30 min at 37 °C. The excess dye was carefully removed using PBS solution, microtiter plates were fixed with 95% ethanol for 15 min at 37 °C, and the sample’s absorbance was measured with a spectrophotometer at 595 nm. To determine the percentage inhibition, the formula [(OD (control) − OD (test)/OD (control)] × 100 was used. *C. albicans* (MTCC 227) was used as a standard strain, and amphotericin-B was used as a positive control.

### Viability of planktonic and sessile cells

The SDB was used to adjust an overnight *C. albicans* culture to a final concentration of 5 × 10^6^ cells/ml. The culture was then incubated for 3 h at 37 °C with agitation, either with β-carotene (200 µg/ml)-containing SDB or with 10% DMSO-containing SDB as a control. Following their centrifugation, the cell pellets were resuspended in 20 µl of SDB. Next, 2 µl of a 0.1% methylene blue solution was combined with 5 µl of the cell suspension on a slide glass, and a cover slip was placed over the mixture. A LM (LABOMED, CXL, USA) was used to view the cell morphology and viability at a magnification of 1000-x [33]. In case of viability detection of sessile cells, as previously reported by Thieme et al. the number of live fungal cells was counted in the presence and absence of β-carotene (100 and 200 µg/ml) in order to ascertain the impact of β-carotene on the viability of sessile cells of *C. albicans* within pre-established biofilms [34]. In summary, following the above-mentioned biofilm formation of *C. albicans* (CA 2, 3, 5, 6, 8, 14, 17, 20, and MTCC 227) using the microtiter plate method, both with β-carotene and with 10% DMSO as a control, the microtiter plate wells were twice washed with PBS to remove any loosely attached cells. After using a pipette tip to scrape the biofilms out of the wells, 200 µl of PBS was added to each well, and the biofilms were then homogenized using a vortex. After the biofilm was homogenized, it was serially diluted with PBS. About 1 ml of each dilution was plated onto a petri dish, and then the molten SDA was poured to count the colony-forming unit (CFU) after a 48-h incubation period at 37 °C. Furthermore, on a slide glass, a volume of 5 µl of the homogenized and diluted biofilms, both treated and untreated, was combined with 2 µl of a 0.1% methylene blue solution, and a cover slip was positioned on top of the mixture. The cell viability was examined using a LM at a 1000-x magnification [33]. (Amphotericin-B was used as a positive control).

### Effect of β-carotene on polysaccharide matrix using phenol sulfuric acid assay

The impact of β-carotene on exopolysaccharide (EPS) was evaluated in accordance with El Zawawy et al. [23]. A 6-well microplate was filled with 2 ml of a prepared *C. albicans* culture in SDB + 1% glucose (10^6^ cells/ml), containing both β-carotene (0.25 and 0.5 MICs) or 10% DMSO as a negative control. The non-adherent cells in the treated and untreated biofilms were removed after a 24-h incubation period at 37 °C. A volume of 2 ml of 0.9% NaCl was then added to the wells, and they were thoroughly cleaned. Following that, sterile test tubes containing 1 ml of 5% phenol were filled with 1 ml of cell suspensions in 0.9% NaCl. After adding 5 ml of 96% sulphuric acid and letting it stand in the dark for 1 h, a UV-Vis spectrophotometer was used to detect the absorbance at 490 nm (Genesys™ 10 S, Thermo Scientific, USA). *C. albicans* (MTCC 227) was used as a standard strain, and amphotericin-B was used as a positive control. The percentage of reduction in EPS production was calculated by using the following formula;

$$\begin{aligned} &\mathrm{Percentage}\;\mathrm{of}\;\mathrm{reduction}\;\mathrm{in}\;\mathrm{EPS}\;\mathrm{production}\;\\&=\;\frac{\mathrm{OD}\left(\mathrm{untreated}\right)\;-\;\mathrm{OD}\left(\mathrm{treated}\right)}{\mathrm{OD}\;\left(\mathrm{untreated}\right)}\;\times100\end{aligned}$$

$$\mathrm{OD}\;(\mathrm{untreated})\;=\;\mathrm{OD}\;\mathrm{at}\;490\;\mathrm{nm}\;\mathrm{of}\;\mathrm{untreated}\mathit\;C\mathit.\mathit\;albicans.$$

$$\begin{aligned} \mathrm{OD}\;(\mathrm{treated})\;=\;&\mathrm{OD}\;\mathrm{at}\;490\;\mathrm{nm}\;\mathrm\beta-\mathrm{carotene}\\&-\mathrm{treated}\;\mathrm{or}\;10\%\;\mathrm{DMSO}\\&-\mathrm{treated}\;C\mathit.\mathit\;albicans. \end{aligned}$$

The statistical analysis was performed by comparing the percentage of reduction in EPS production by *C. albicans* after treatment with β-carotene (0.25 and 0.5 MICs) with the percentage of reduction of EPS by *C. albicans* after treating with 10% DMSO.

### Effect of β-carotene on hyphae-specific gene by quantitative Reverse Transcription Polymerase Chain Reaction (qRT-PCR)

The influence of β-carotene on the expression of the hypha-specific gene, *ALS3*, was assessed by qRT-PCR. *C. albicans* suspension in RPMI-1640 medium (10^6^ CFU/ml) was treated with 0.5 MIC of β-carotene under shaking conditions for 4 h at 37 °C, where 10% DMSO served as a control. The fungal cells were washed, and the total RNA was extracted employing TRIzol reagent (Invitrogen, Carlsbad, CA, USA), and the cDNA was synthesized via TransScript^®^ First-Strand cDNA Synthesis SuperMix (TransGen, Beijing, China). Primers for the *ALS3* gene and the housekeeping internal control gene, the *ACT1* gene, were listed in Table S1. The following parameters were used for qRT-PCR using Rotor-Gene Q (Qiagen, USA): initial denaturation (3 min/95°C), followed by 40 cycles of denaturation (1 min/95°C), annealing (30 s/58°C), and extension (20 s/72°C). The CT value of the *ALS3* gene was normalized to the CT value of the *ACT1* gene, and the relative gene expression was determined using the 2^−ΔΔCT^ assay [35]. (Amphotericin-B was used as a positive control)

### Detecting the interaction of β-carotene with agglutinin-like protein 3 (Als3) using molecular docking analysis

The crystallographic structure of the Als3 protein (PDB: 4LEE) was retrieved from the Protein Data Bank (https://www.rcsb.org/structure/4LEE), and the β-carotene structure was downloaded from PubChem (https://pubchem.ncbi.nlm.nih.gov/compound/5280489). After energy minimization and preparation of Als3 protein and β-carotene via the Autodock Tools (ADT) software, the AutoDock Vina program was utilized to execute molecular docking to assess the binding affinities of β-carotene with Als3 protein [36, 37].

### Assessment of anticandidal activity of β-carotene against MDR C. albicans-infected diabetic wounds in a rat model

Anticandidal action of β-carotene against MDR *C. albicans* was studied using a wound infection model in diabetic rats, and the Research Ethics Committee of the Faculty of Pharmacy at Tanta University (TP/RE/2/25p-02) allowed the model steps in accordance with ARRIVE guidelines [6, 38].

- Step 1: A total of 20 male Sprague–Dawley rats, aged between 10 and 12 weeks, weighing between 300 and 400 g, were obtained from the animal house of the Faculty of Pharmacy at Tanta University (Tanta, Egypt), and the rats were categorized into 2 groups (control and treated).
- Step 2: Rats were kept separately in vented cages with 12 h of light and 12 h of darkness, at room temperature, and with unhindered access to food and drink to adjust to their surroundings.
- Step 3: Diabetes was produced in rats after 7 days of acclimatization with an intraperitoneal injection of streptozocin (STZ) (Sigma-Aldrich) at a dosage of 50 mg/kg for two consecutive days.
- Step 4: Following 48 h of the second injection, the blood glucose level was measured after being drawn from the tail vein, and the rats were identified as diabetic when their glucose level exceeded 200 mg/dL.
- Step 5: Rats that had been anesthetized with ketamine (40 mg/kg) and xylazine (5 mg/kg) were shaved from the back side and sterilized with 10% povidone-iodine. After that, the excisional wound (10 mm thick) was created on the rat’s dorsal portion using biopsy punches.
- Step 6: The MDR *C. albicans* (CA 14) cell suspension (5 × 10^7^ CFU/ml) was subcutaneously injected (100 µl) into the diabetic wounds for two consecutive days.
- Step 7: On the next day, the control group was subcutaneously administered 20 µl of the vehicle (10% DMSO as a negative control), and β-carotene (200 µg/ml) was subcutaneously injected into the treated group.
- Step 8: A sterile ruler was used to determine the wound size at day 0, 4, 8, and 12, and the wound healing ratios were calculated using the formula below.
  $$\begin{aligned} &\mathrm{Wound}\;\mathrm{healing}\;\mathrm{ratio}\;\\&=\frac{\mathrm{Initial}\;\mathrm{wound}\;\mathrm{size}\;-\mathrm{the}\;\mathrm{current}\;\mathrm{wound}\;\mathrm{size}\;\mathrm{measured}\;\mathrm{by}\;\mathrm{ruler}}{\mathrm{Initial}\;\mathrm{wound}\;\mathrm{size}}\;\\&\times\;100 \end{aligned}$$
- Step 9: On day 12 of the experiment, rats from each group were put to death by CO_2_ inhalation, and the skin lesions (2 to 5 mm) were removed.
- Step 10: After the wound samples were collected, they were preserved in a 10% buffered formalin solution, embedded in a paraffin block, sectioned at a thickness of 5 m, and stained with hematoxylin and eosin (H & E) for histological examination.
- Step 11: Tissues were homogenized using a sonicator, and the resulting suspensions were serially diluted and plated on SDA to determine the fungal count. The following formula was used to determine the tissue’s fungal burden:
  $$\begin{aligned} \mathrm{CFU}/\mathrm g=\left(\mathrm{plate}\;\mathrm{count}\times\frac1{dilution}\times10\right)/\mathrm{weight}\;\mathrm{of}\;\mathrm{homogenized}\;\mathrm{tissue} \end{aligned}$$

### Statistical analysis

The data were reported as mean ± SD, and all experiments were run in triplicate, except for AFST. GraphPad Prism version 5 software was utilized to compare the two groups using a *t*-test. The p-value < 0.05 served as a significance level.

## Results

### Identification of C. albicans isolates

**Fig. 1:**
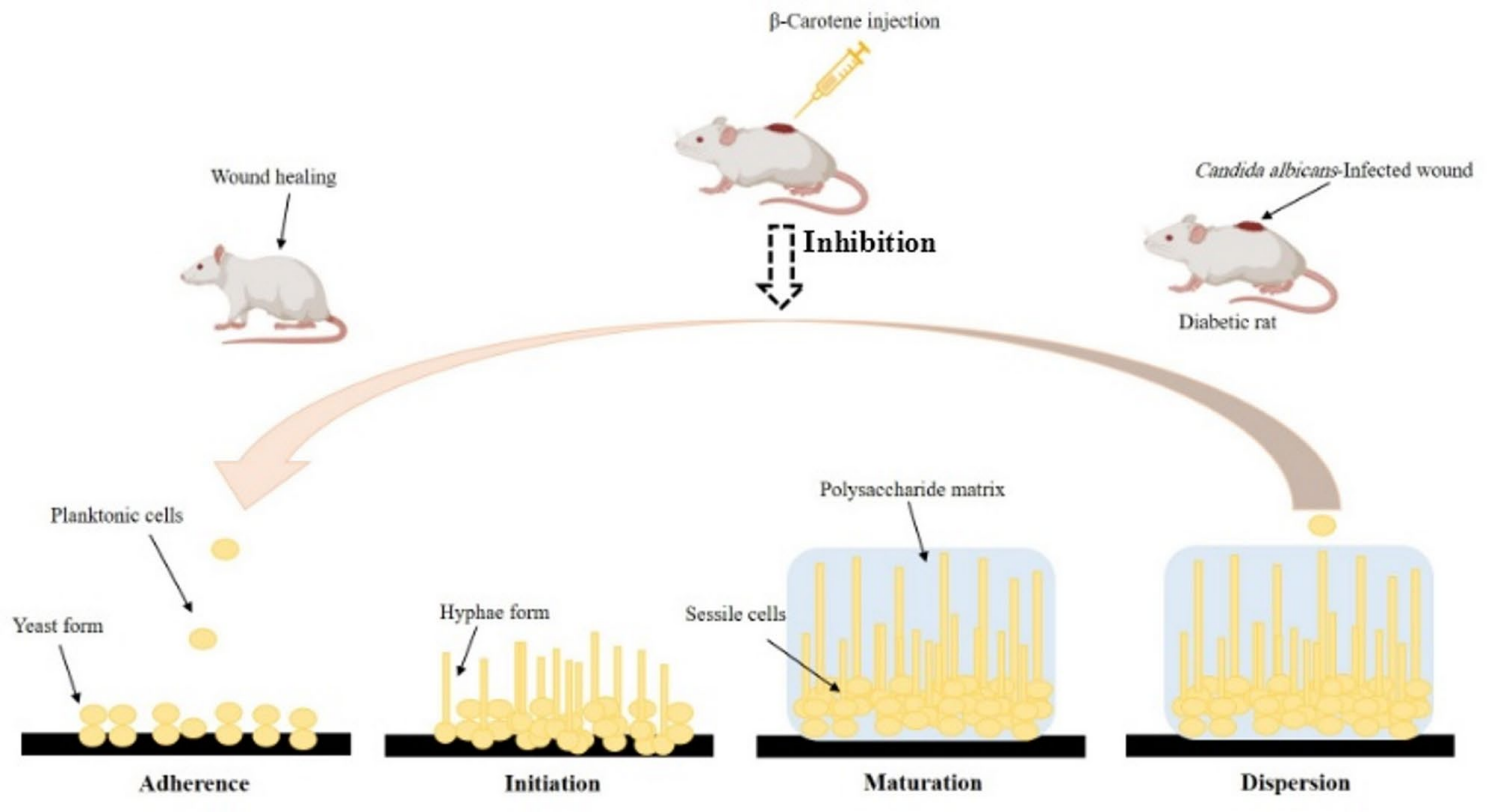
A graphical abstract shows the mechanism of action of β-carotene; β-carotene induced wound healing in diabetic rats via disrupting biofilms of MDR *C. albicans* through inhibiting adherence, initiation, and maturation stages

A total of 20 *C.* albicans were identified according to their colony morphology on SDA, which were creamy, smooth, and pasty convex colonies, and their Gram-staining results. As well, the isolated colonies displayed green colonies on the colony color on CHROMagar™ Candida medium, and a germ tube when investigated under LM. Additionally, the identification was further confirmed by MALDI-TOF MS (Fig. S1) (Fig. 1).

### The AFST for C. albicans and MDR isolates detection

According to the AFST results, the isolates of C. albicans exhibited a susceptibility pattern of 8 resistant and 12 susceptible to miconazole and 6 resistant and 14 susceptible to itraconazole. The majority of isolates were sensitive to fluconazole (19 susceptible and 1 resistant). The susceptibility pattern for nystatin was 12 susceptible and 8 resistant isolates. All isolates were susceptible to amphotericin-B, and C. albicans isolates CA 2, 3, 5, 6, 8, 14, 17, and 20 were classified as MDR, as these isolates were resistant to at least one antifungal agent from two separate drug classes (Table 1). *C. albicans* MTCC 227 was sensitive to all tested antifungal agents except for fluconazole.

**Table 1:** Susceptibility pattern of *C. albicans* isolates (*n* = 20) to standard anti-candida drugs

Isolates	Zone diameter (mm)/Zone interpretation (S or R)				
	MIC (30 µg)	AMB (100 µg)	NS (50 IU)	FLC (10 µg)	IT (10 µg)
CA 1	24/S	30/S	28/S	27/S	26/S
CA 2^#^	10/R	22/S	9/R	22/S	12/R
CA 3^#^	11/R	24/S	9/R	25/S	24/S
CA 4	26/S	29/S	28/S	28/S	23/S
CA 5^#^	10/R	23/S	9/R	23/S	10/R
CA 6^#^	11/R	25/S	9/R	23/S	27/S
CA 7	21/S	30/S	29/S	26/S	30/S
CA 8^#^	11/R	22/S	9/R	24/S	25/S
CA 9	20/S	29/S	26/S	28/S	25/S
CA 10	32/S	32/S	26/S	30/S	30/S
CA 11	27/S	30/S	27/S	30/S	12/R
CA 12	25/S	28/S	30/S	29/S	29/S
CA 13	22/S	32/S	29/S	29/S	25/S
CA 14^#^	10/R	21/S	9/R	12/R	9/R
CA 15	24/S	29/S	26/S	28/S	30/S
CA 16	29/S	29/S	28/S	26/S	26/S
CA 17^#^	10/R	24/S	9/R	21/S	12/R
CA 18	30/S	28/S	30/S	25/S	26/S
CA 19	23/S	30/S	30/S	28/S	24/S
CA 20^#^	10/R	24/S	9/R	22/S	11/R
*C. albicans* MTCC 227	23/S	31/S	29/S	12/R	28/S

S indicates sensitive to the tested antifungal agent, and R indicates resistant to the tested antifungal agent

*MIC* miconazole, *AMB* amphotericin-B, *NS* nystatin, *FLC* fluconazole, *IT* itraconazole

^**#**^ indicates multidrug-resistant isolates (resistant to at least one antifungal agent from two separate drug classes). Isolates are sensitive to MIC when zone diameter ≥ 20 mm and are considered resistant when zone diameter ≤ 11 mm. Isolates are sensitive to AMB when zone diameter ≥ 15 mm and are considered resistant when zone diameter ≤ 9 mm. Isolates are sensitive to NS when zone diameter ≥ 15 mm and are considered resistant when zone diameter ≤ 9 mm. Isolates are sensitive to FLC when zone diameter ≥ 19 mm and are considered resistant when zone diameter ≤ 14 mm. Isolates are sensitive to IT when zone diameter ≥ 23 mm and are considered resistant when zone diameter ≤ 13 mm

### β-Carotene inhibited the viability of C. albicans isolates

**Fig. 2:**
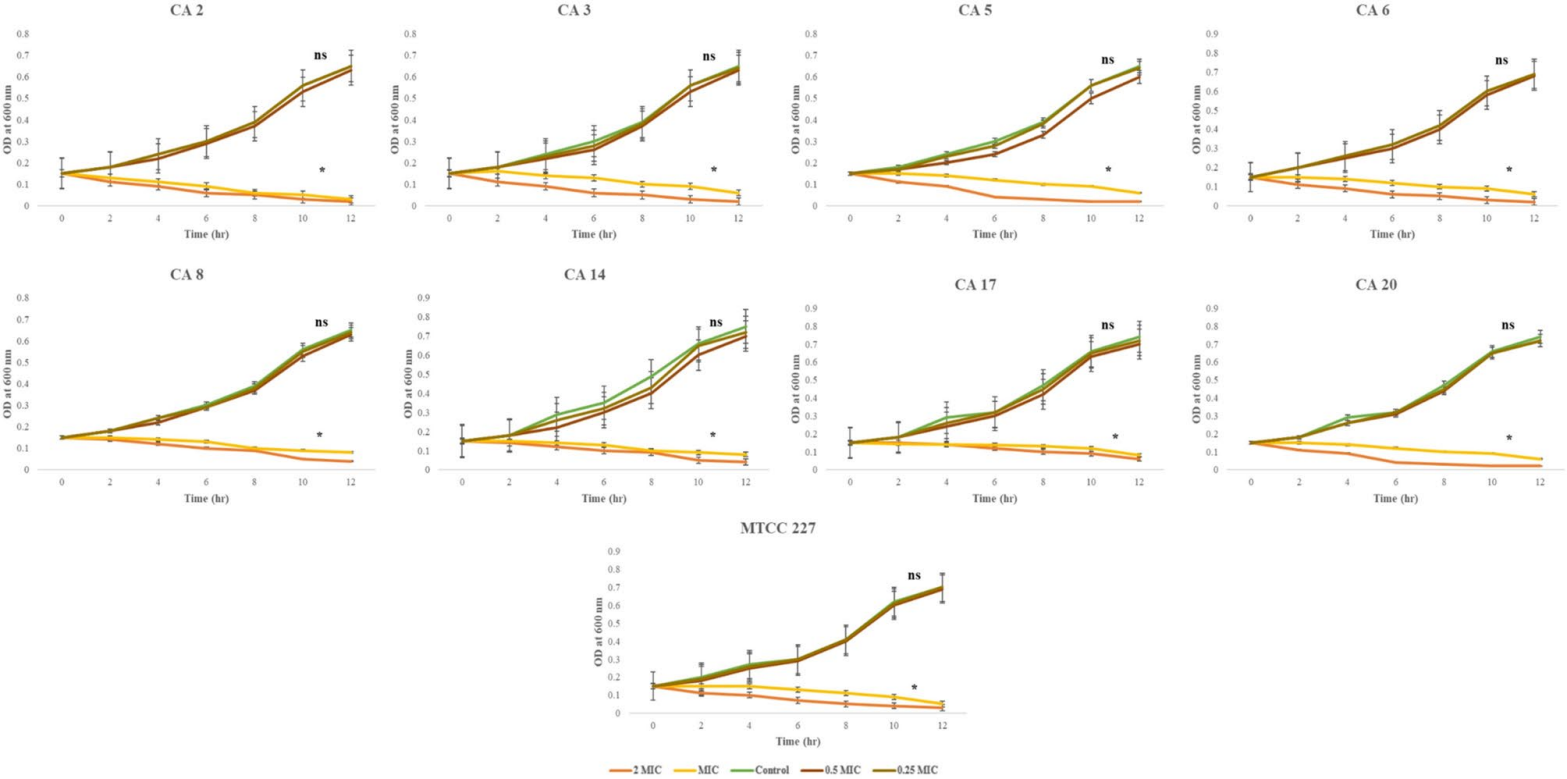
Growth kinetics of MDR *C. albicans* (*n* = 8) and *C. albicans* MTCC 227 in the presence of 0, 0.25, 0.5, 1, and 2 MICs of β-carotene. Culture growth was monitored at 2 h intervals through estimation of OD at 600 nm. The graph shows the average values of triplicate measurements and the error bars indicate standard deviations. The asterisk signifies the significance at *P* < 0.05 (for MIC and 2 MIC), and ns indicates non-significance (for 0.25 MIC and 0.5 MIC) when compared to control

The effect of β-carotene (200 µg/ml) on the *C. albicans* viability was determined utilizing the well diffusion assay. The data revealed that the growth of all *C. albicans* isolates was significantly (*P* < 0.05) inhibited by β-carotene, and the resultant inhibition zones ranged from 34 mm to 14 mm (Fig. S2 A) (Table 2). The positive control amphotericin-B showed inhibition zones ranging from 23 mm to 36 mm. The MIC of β-carotene was also determined using the microplate dilution method, and the plate was visualized against a black background. The MICs of β-carotene were 100 µg/ml for all isolates except CA 1, CA 4, and CA 5, which were 50 µg/ml, and the MIC of β-carotene against *C. albicans* MTCC 227 was 100 µg/ml (Fig. S2 B) (Table 2). The MICs of amphotericin-B against all tested isolates were 0.25 and 0.5 µg/ml. In addition, the effect of β-carotene on the viability of *C. albicans* was confirmed by methylene blue staining, which stained dead cells blue, and the live cells remained unstained. It was observed that control cells remained live, containing no stain, while the treated cells stained blue, which proved their death via β-carotene (Fig. S2 C, D). Moreover, examination of the growth kinetics of MDR *C. albicans* (CA 2, 3, 5, 6, 8, 14, 17, and 20) and *C. albicans* MTCC 227 in the presence of β-carotene demonstrated that the 0.25 and 0.5 MIC curves exhibited an analogous shape to the control curve, with a slight downshift in the OD values compared to the control. Each of the 1 and 2 MICs of β-carotene exhibited antifungal action during the 12-h duration of the experiment, showing a great drop in OD values compared to the control (Fig. 2**)**. These interpretations correlate with the displayed anticandidal activity of β-carotene by well diffusion and broth microdilution methods and emphasize the powerful anticandidal activity of β-carotene.

**Table 2:** Inhibition zones and MIC values of β-carotene against *C. albicans* isolates

	β-carotene					
Isolates	**Inhibition zones (mm)**	**MIC** **µg/ml**	**Isolates**	**Inhibition zones (mm)**		**MIC** **µg/ml**
CA 1	14 ± 1*	50 ± 0	CA 11		22 ± 0*	100 ± 0
CA 2	32 ± 2*	100 ± 0	CA 12		20 ± 2*	100 ± 0
CA 3	31 ± 1*	100 ± 0	CA 13		20 ± 0*	100 ± 0
CA 4	15 ± 3*	50 ± 0	CA 14		21 ± 1*	100 ± 0
CA 5	17 ± 0*	50 ± 0	CA 15		19 ± 2*	100 ± 0
CA 6	23 ± 1*	100 ± 0	CA 16		27 ± 0*	100 ± 0
CA 7	25 ± 0*	100 ± 0	CA 17		22 ± 1*	100 ± 0
CA 8	20 ± 2*	100 ± 0	CA 18		28 ± 2*	100 ± 0
CA 9	29 ± 2*	100 ± 0	CA 19		34 ± 2*	100 ± 0
CA 10	21 ± 1*	100 ± 0	CA 20		26 ± 0*	100 ± 0
*C. albicans* MTCC 227	24 ± 0*	100 ± 0				

Values indicate the mean ± SD (*n* = 20)

* indicates statistical significance (P value > 0.001) in comparison with the control (10% DMSO) that had no inhibition zone against all tested isolates

### Strong biofilm-forming C. albicans isolates

The CV staining method was used to assess *C. albicans’* (*n* = 20) capacity to produce biofilms, and the OD was assessed at 595 nm. According to Table 3, CA 2, 3, 5, 6, 8, 14, 17, 20, and *C. albicans* MTCC 227 had ODs that were higher than 4 x ODC, demonstrating their strong capacity to produce biofilms, and these isolates were utilized in the subsequent experiments.

**Table 3:** Classification of *C. albicans* biofilms based on OD measurements at 595 nm

Isolates	Average OD_595 nm_	Biofilm type	Isolates	Average OD_595 nm_	Biofilm type
CA 1	0.34 ± 0.017	Moderate	CA 11	0.371 ± 0.09	Moderate
CA 2	0.834 ± 0.02	Strong	CA 12	0.26 ± 0.051	Moderate
CA 3	0.798 ± 0.14	Strong	CA 13	0.336 ± 0.047	Moderate
CA 4	0.16 ± 0.08	Weak	CA 14	0.956 ± 0.17	Strong
CA 5	1.13 ± 0.113	Strong	CA 15	0.183 ± 0.08	Weak
CA 6	0.826 ± 0.095	Strong	CA 16	0.381 ± 0.06	Moderate
CA 7	0.392 ± 0.042	Moderate	CA 17	1.32 ± 0.22	Strong
CA 8	0.842 ± 0.11	Strong	CA 18	0.192 ± 0.037	Weak
CA 9	0.291 ± 0.032	Moderate	CA 19	0.175 ± 0.024	Weak
CA 10	0.182 ± 0.06	Weak	CA 20	0.982 ± 0.58	Strong
*C. albicans* MTCC 227	0.89 ± 0.02	Strong			

Values indicate the mean ± SD (*n* = 20)

The ODs of weak biofilm-forming *C. albicans* ranged from 0.12 to 0.24, while the ODs of moderate biofilm-forming isolates ranged from 0.24 to 0.48, and the ODs of strong biofilm-forming isolates were ≥ 0.48

### Biofilm eradication of MDR strong biofilm-forming C. albicans via β-carotene from polystyrene plates and silicon catheters

**Fig. 3:**
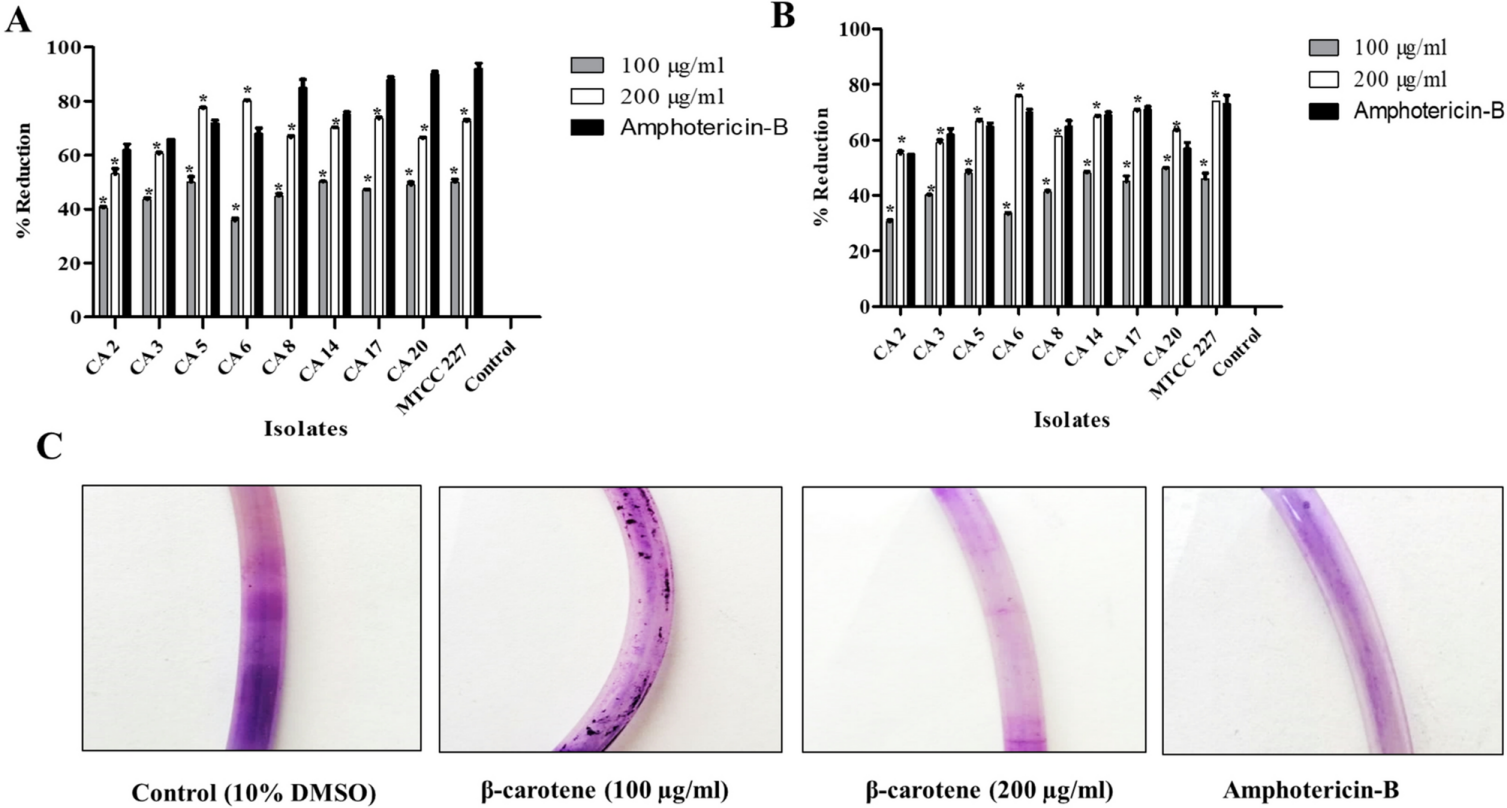
Eradication of mature biofilm of MDR *C. albicans* (*n* = 8) and *C. albicans* MTCC 227 by β-carotene. The bar chart represented a dose-dependent biofilm-eradicating activity of β-carotene against *C. albicans* biofilms on polystyrene plates (**A**). The bar chart represented a dose-dependent biofilm-eradicating activity of β-carotene against *C. albicans* biofilms on silicon catheters (**B**). Illustrative images demonstrated the disruption of pre-established *C. albicans* biofilm (CA 14) on silicon catheter after treatment with 100 and 200 µg/ml of β-carotene (**C**). The graphs show the average values of triplicate measurements for each isolate and the error bars indicate standard deviations. The asterisk signifies the significance at *P* < 0.001. The 10% DMSO acts as a negative control, and amphotericin-B acts as a positive control

Using CV staining, the activity of β-carotene on the mature strong biofilms of *C. albicans* (*n* = 8) was evaluated via microtiter plate assay, and the percentage decrease in the detected ODs at 595 nm was estimated. β-Carotene significantly (*P* *< 0.05*) eliminated the biofilm of all tested *C. albicans* in a dose-dependent manner. As shown in Fig. 3A, the biofilm eradication ability ranged from 50 to 70% at a concentration of 100 µg/ml and from 80 to 95% at a concentration of 200 µg/ml. Whereas, amphotericin-B showed an antibiofilm eradication activity against *C. albicans* ranging from 62% to 92%. The CA 17’s biofilm, the strongest biofilm, was eradicated by 47.2% and 73.5% after treatment with 100 and 200 µg/ml of β-carotene, respectively, and *C. albicans* MTCC 227’s biofilm was eradicated by 50% and 72.6% after treatment with 100 and 200 µg/ml of β-carotene, respectively. Using a silicone catheter as a model for medical devices, the biofilm eradication efficacy of β-carotene against *C. albicans* biofilms was assessed. The untreated *C. albicans* built up a dense biofilm on silicon catheters; in contrast, the β-carotene-treated *C. albicans* showed thin biofilms after staining with CV. The percentages of eradication of biofilm ranged from 30.6 to 49.8% after treatment with 100 µg/ml of β-carotene, where treating with 200 µg/ml of β-carotene eradicated biofilm with a percentage of 55 to 75.9%, as illustrated in Fig. 3B, C. The percentage of *C. albicans* biofilm eradication by amphotericin-B ranged from 55% to 73% in a silicon catheter model. The percentages of eradication of CA 17’s biofilm from silicon catheters were 45% and 70.5% after treating with β-carotene at concentrations of 100 and 200 µg/ml, respectively. While *C. albicans* MTCC 227’s biofilm was eradicated from silicon catheters by percentages of 46% and 74% when treated with 100 and 200 µg/ml of β-carotene, respectively.

### Detection of biofilm eradication by simple staining and dual staining approaches

**Fig. 4:**
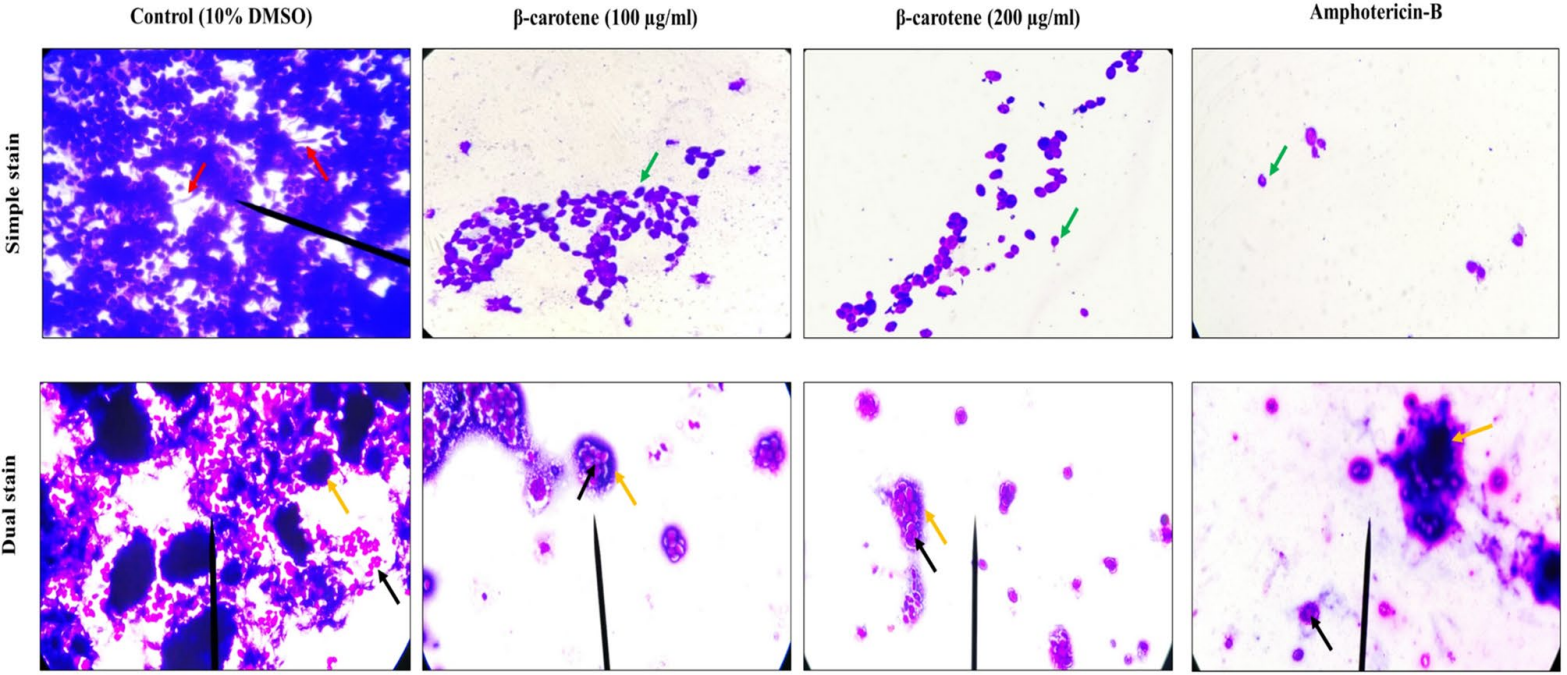
Representative images of LM at 1000-x magnification demonstrated the antibiofilm activity of β-carotene against *C. albicans* (CA 14) at a concentration of 100 and 200 µg/ml. The simple staining technique showed the disrupted *C. albicans* mature biofilm with no hyphae formation, and a few yeast cells (green arrows). Amphotericin-B (positive control) showed a disrupted *C. albicans* biofilms; on the other hand, the 10% DMSO-treated (negative control) biofilm of *C. albicans* showed dense biomass and hyphae formation (Red arrows). Dual staining technique showed a reduction in polysaccharide matrix (blue color/orange arrows) and biofilm-forming cells (magenta-red/black arrows) in *C. albicans* biofilm via β-carotene treatment. The application of amphotericin-B also reduced the biofilm-forming cells with negligible effect on polysaccharide matrix, in contrast, the negative control biofilm showed a thick polysaccharide matrix and a high number of biofilm-forming fungal cells

The LM observations provided valuable data on the effect of β-carotene on biofilm biomass. Treatment with β-carotene disrupted the biomass of the biofilms of CA 2, 3, 5, 6, 8, 14, 17, 20, and MTCC 227 strains, showing a decrease in the number of cells within the biofilms and a loss of connection between cells within the biofilms. As well as, the application of amphotericin-B showed the same antibiofilm activity of β-carotene against *C. albicans*. In addition, LM images of control biofilms showed the presence of a dense complex structure of biofilms, having hyphae and yeast cells. In contrast, the presence of 100 and 200 µg/ml of β-carotene reduced the biomass of biofilms in all tested strains with complete hyphal disappearance and consisted mostly of yeast cells (Fig. 4). When the dual-staining approach was used to visualize the structure of the biofilm, the polysaccharide layer of the biofilm turned dark blue. At the same time, the fungal cells were dyed magenta-red by acid fuchsin, which made it possible to distinguish the yeast cells from the biofilm matrix. As displayed in Fig. 4, the treatment of *C. albicans* biofilms (CA 2, 3, 5, 6, 8, 14, 17, 20, and MTCC 227) via β-carotene led to eradication of their biofilms by reducing the number of biofilm-forming fungal cells and lowering the polysaccharide matrix of *C. albicans* biofilms. In addition, the treatment by amphotericin-B led to decrease the number of *C. albicans* without showing a significant effect of polysaccharide matrix.

### Detection of biofilm eradication by confocal laser scanning microscope

**Fig. 5:**
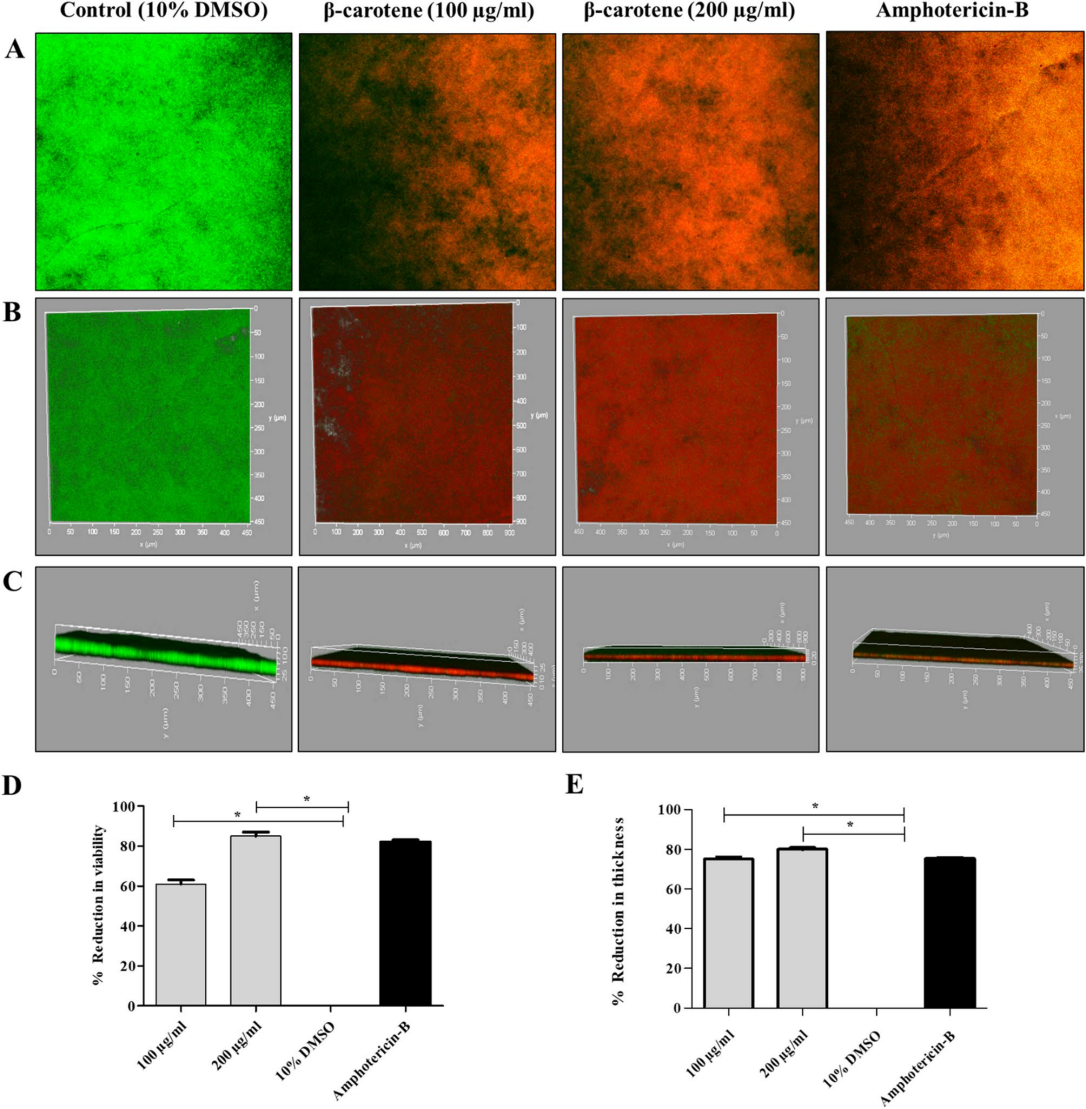
Screening biofilm eradication activity of β-carotene against mature biofilm of *C. albicans* (CA 14) at a concentration of 100 and 200 µg/ml using CLSM. The two-dimensional CLSM images showing the effect of β-carotene on cell viability, the viable cells emitted green fluorescence, and the dead cells emitted red fluorescence (**A**). The 3D top-view CLSM images showed the effect of β-carotene on cell viability; the treated biofilms emitted red fluorescence of dead fungal cells (**B**). The 3D side-view CLSM images presenting the outcome of β-carotene on biofilm thickness (**C**). The bar chart represented the percentage of reduction in cell viability by β-carotene (**D**). The bar chart represents β-carotene-induced reduction in biofilm thickness (**E**). The graph shows the average values of triplicate measurements and the error bars indicate standard deviations. The asterisk signifies the significance at *P* < 0.001. The 10% DMSO acts as a negative control, and amphotericin-B acts as a positive control

The antibiofilm effect of β-carotene on *C. albicans* biofilms was further confirmed by CLSM examination (Fig. 5). To analyze the effect of β-carotene on the viability of cells within the mature biofilms of *C. albicans* (CA 14), the cells were stained with PI and AO for staining the viable and dead cells, respectively. The intensities of the red color of PI and the green color of AO were measured, and the obtained data showed a significant (*P* < 0.05) reduction, 61% for 100 µg/ml of β-carotene and 85% for 200 µg/ml of β-carotene, in the cell viability in the treated CA 14’s biofilm compared to the control one. While treating with amphotericin-B decreased the viability of sessile cells by 82%.

To examine the effects of β-carotene on biofilm thickness, three-dimensional (3D) images were taken for the control and treated samples, and Z-stacks were prepared to estimate the difference in the thickness. The thickness of the CA 14’s mature biofilm was 100 μm, and it became 25 μm after treatment with β-carotene (100 µg/ml) and amphotericin-B, whereas, it became 20 μm after treatment with β-carotene (200 µg/ml).

### β-Carotene inhibited C. albicans adherence to polystyrene plates

**Fig. 6:**
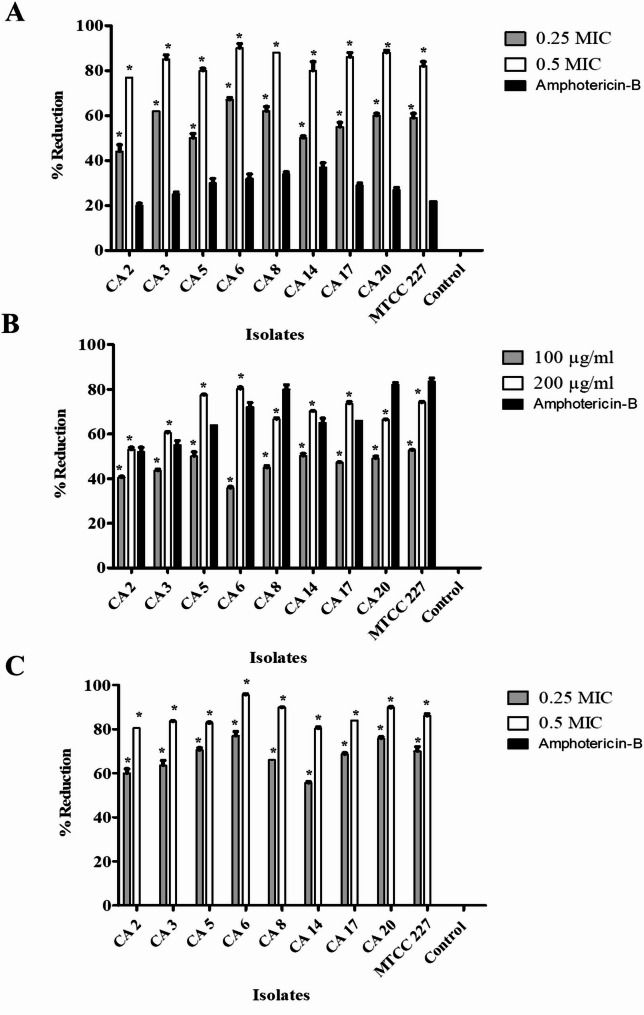
Antibiofilm mechanism of β-carotene against MDR C. albicans (n = 8) and C. albicans MTCC 227 by targeting adherence, sessile cell viability, and polysaccharide matrix production. The bar chart represented the percentages in reduction of *C. albicans* adherence to polystyrene plates after treatment with 0.25 and 0.5 MICs of β-carotene (**A**). The bar chart represented the percentage of reduction in viability of sessile cells after treatment with 100 and 200 µg/ml of β-carotene (**B**). The bar chart represented the percentage of reduction in polysaccharide matrix after treatment with 0.25 and 0.5 MICs of β-carotene (**C**). The graphs show the average values of triplicate measurements for each isolate and the error bars indicate standard deviations. The asterisk signifies the significance at P < 0.001. The 10% DMSO acts as a negative control, and amphotericin-B acts as a positive control

**Fig. 7:**
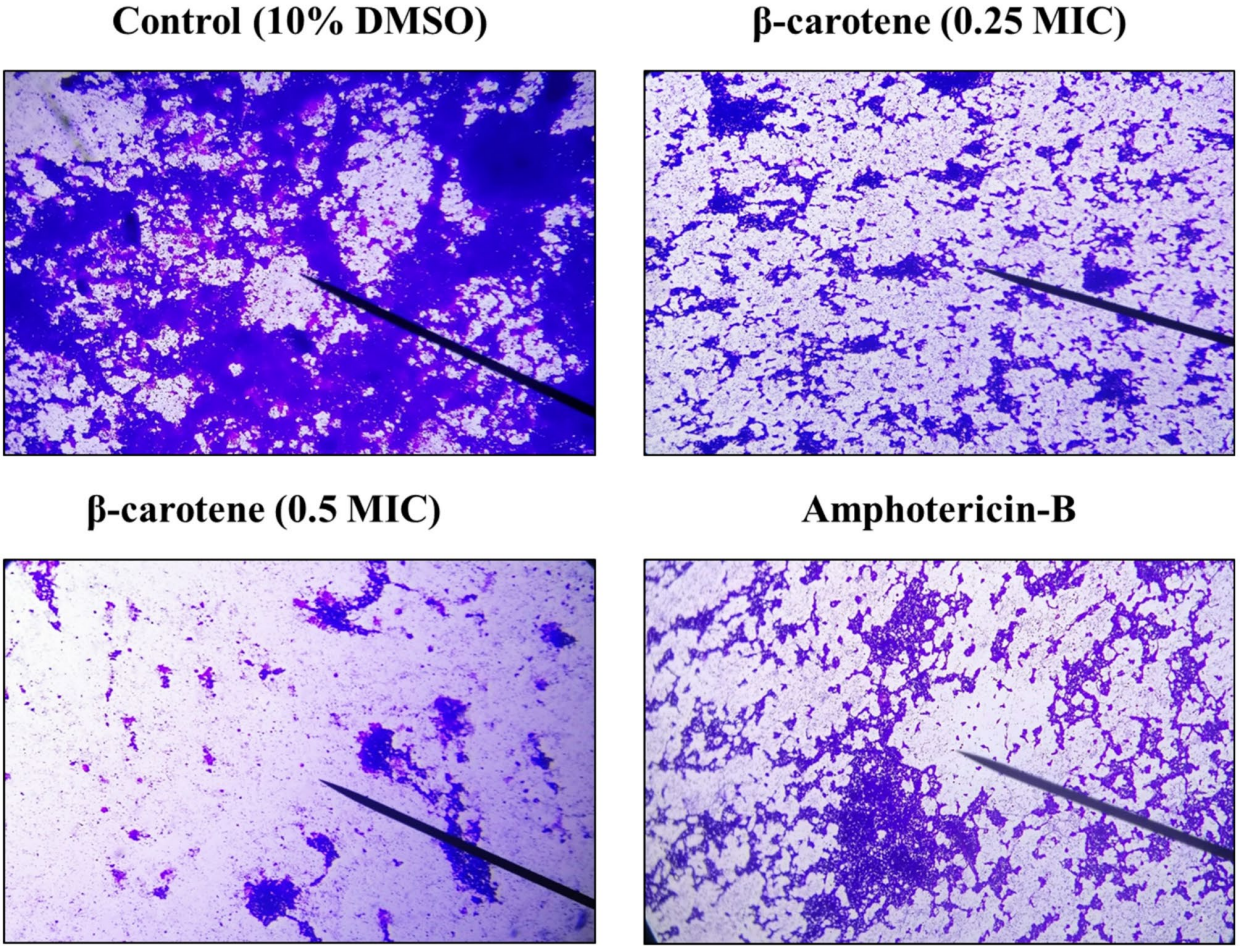
Representative images of LM at 100-x magnification displayed the reduction in adherence of *C. albicans* cells (CA 14) after treatment with 0.25 and 0.5 MICs of β-carotene, showing a lesser crystal violet-stained adherent biomass. The 10% DMSO acts as a negative control, and amphotericin-B acts as a positive control

The adhesion assay was carried out using the CV staining approach. As presented in Fig. 6A, the adherence of *C. albicans* to polystyrene plates was suppressed by 44% to 67% after treatment with 0.25 MIC of β-carotene, while treatment with 0.5 MIC of β-carotene reduced the adherence up to 90%. As well, the application of amphotericin-B as a positive control led to 20% to 37% reduction in adherence of fungal cells. The 0.25 and 0.5 MICs of β-carotene reduced the adherence of CA 17 isolate by percentages of 55% and 86%, respectively, and reduced the adherence of *C. albicans* MTCC 227 by percentages of 59% and 82%, respectively. Additionally, the adherent biomass was inspected via LM, which emphasized the anti-adherent action of β-carotene against *C. albicans*. The untreated *C. albicans* showed a massive adherent biomass; in contrast, *C. albicans* revealed little adherent biomass after treatment with β-carotene (0.25 and 0.5 MICs) and amphotericin-B, however, the anti-adherent effect of amphotericin-B was less than β-carotene (Fig. 7).

### β-Carotene diminished the viability of sessile cells of C. albicans biofilms

The influence of β-carotene on the viability of sessile cells in pre-established *C. albicans* biofilms was investigated by measuring viable bacterial count and staining with methylene blue. Our data showed that β-carotene significantly (*P* < 0.05) lowered the *C. albicans* viability by 35.8 to 50.2% and 53 to 80% at concentrations of 100 and 200 µg/ml, respectively. The percentages of reduction in the viability of sessile cells in CA 17’s biofilm were 47.2% and 73.5% when treated with 100 and 200 µg/ml of β-carotene, respectively, and the treatment of *C. albicans* MTCC 227’s biofilm with 100 and 200 µg/ml of β-carotene decreased the viability of sessile cells by 52.7% and 74%, respectively (Fig. 6B). The positive control (amphotericin-B) showed 52% to 83.5% reduction in viability of sessile cells. After screening of methylene blue-stained *C. albicans*, it was found that β-carotene (100 and 200 µg/ml) and amphotericin-B increased the number of dead blue-stained cells, while the untreated cells showed a smaller number of dead cells, and the remaining cells were unstained and viable.

### β-Carotene reduced the polysaccharide matrix of C. albicans biofilms

The phenol sulfuric acid method was performed to investigate the effect of β-carotene on polysaccharide production by *C. albicans*. Our results showed that the treatment by β-carotene significantly (*P* < 0.05) reduced the amount of polysaccharide matrix in all tested isolated compared to untreated cells. The percentages of reduction in polysaccharide matrix ranged from 55.6 to 77% after treatment with 0.25 MIC of β-carotene, while treatment with 0.5 MIC of β-carotene decreased polysaccharide matrix by 80.3 to 95.6%. In contrast, the application of amphotericin-B manifested no effect on EPS production by *C. albicans*. The percentages of reduction in the production of EPS by CA 17 isolate were 68.6% and 84.01% after treatment with 0.25 and 0.5 MICs of β-carotene, respectively, where treatment of *C. albicans* MTCC 227 with 0.25 and 0.5 MICs of β-carotene repressed the production of EPS by 70% and 86%, respectively (Fig. 6C).

### β-Carotene inhibited the yeast-to-hyphae transition of C. albicans 

The effect of β-carotene on the transition of *C. albicans* from yeast to hyphae was mediated by incubation in RPMI-1640 medium supplemented with 15% FBS serum at 37 °C with and without 0.25 and 0.5 MICs of β-carotene for 4 h. All tested fungi showed yeast budding and yeast-to-hyphae transition in the presence of 10% DMSO (negative control) or amphotericin-B (positive control); in contrast, β-carotene-treated cells were devoid of yeast budding and hyphae formation.

### β-Carotene disrupted the action of agglutinin-like protein 3 (Als3) by inhibiting its expression and formation of inactive complex

**Fig. 8:**
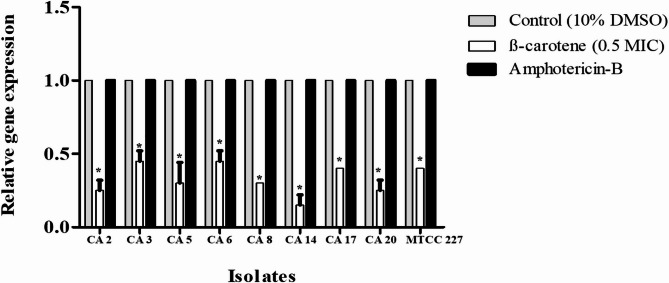
The relative fold gene expression of *ALS3* gene after treatment of *C. albicans* isolates (CA 2, 3, 5, 6, 8, 14, 17, 20, and MTCC 227) with β-carotene (0.5 MIC). The 10% DMSO acts as a negative control, and amphotericin-B acts as a positive control. Results represent the average of two independent experiments ± SD. * represents *P* < 0.05 when compared with the negative control

The effect of β-carotene on the expression profile of the hypha-specific gene, *ALS3*, was analyzed. After incubation of *C. albicans* cells in RPMI-1640 medium at 37 °C for 4 h in the absence or presence of β-carotene (0.5 MIC), the expression of the indicated gene was determined by qRT-PCR. The expression level of the *ALS3* gene was displayed after normalization with the internal control housekeeping gene *ACT1*. Transcripts results revealed that β-carotene significantly (*P* < 0.05) downregulated the expression of the *ALS3* gene, and the percentages of reduction ranged from 55 to 85% (Fig. 8). The percentage of reduction in *ALS3* gene expression in CA 17 isolate and *C. albicans* MTCC 227 was 60% after treatment with 0.5 MIC of β-carotene (relative fold expression = 0.4). On the other hand the negative control (10% DMSO) and amphotericin-B had no effect on the expression *ALS3* gene in all tested isolates (relative fold expression = 1). Additionally, the in silico analysis by molecular docking investigated the effect of β-carotene on the Als3 protein. It was reported that β-carotene bound to Als3 protein through hydrophobic interaction with five amino acids and the binding energy equal to −9.8 kcal/mol (Table 4) (Fig. S3). Collectively, β-carotene inhibited the action of Als3 protein in *C. albicans* through lowering its expression and forming of inactive complex with it.

**Table 4:** Interacting amino acids in the β-carotene and agglutinin-like protein 3 complex

Amino acids	Binding free energy (Kcal/mol)
Threonine (Thr) 142	6.1
Phenylalanine (Phe) 58	1.12
Phe 117	3.66
Cysteine (Cys) 56	2.45
Valine (Val) 116	2.75

### Assessment of the ability of β-carotene to fight C. albicans infection in diabetic wounds using a rat model

**Fig. 9:**
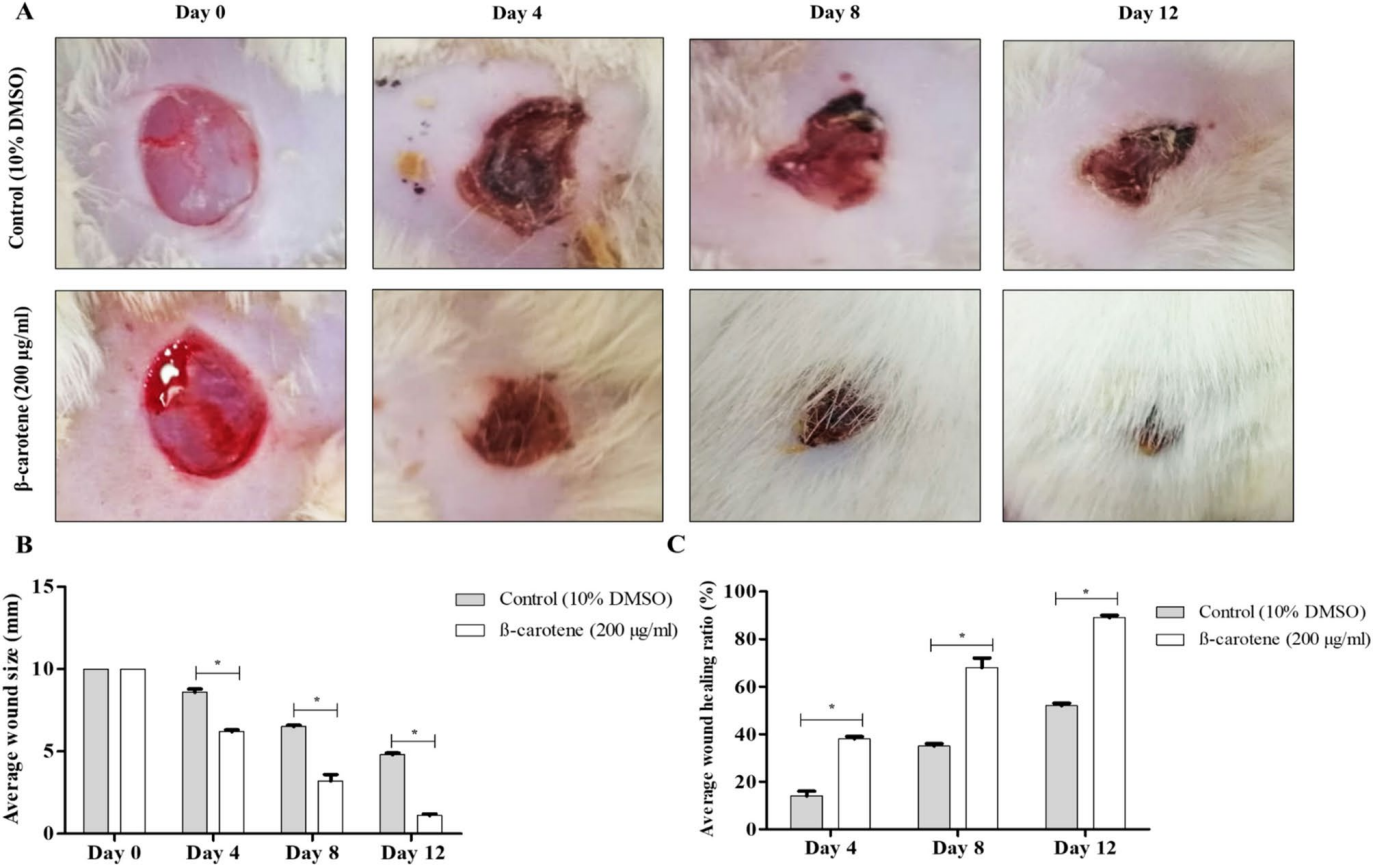
β-Carotene accelerated wound healing in diabetic rats infected with *C. albicans* (CA 14) (number per group = 6). Representative images of *C. albicans*-infected wounds in diabetic rats in control and treated groups showing a time-dependent wound healing process (**A**). The bar chart represented the average wound size per day in the control and treated groups (**B**). The bar chart symbolized the average wound healing ratio per day in the control and treated groups (**C**). The graphs show the average values of triplicate measurements and the error bars indicate standard deviations. The asterisk signifies the significance at *P* < 0.001

**Fig. 10:**
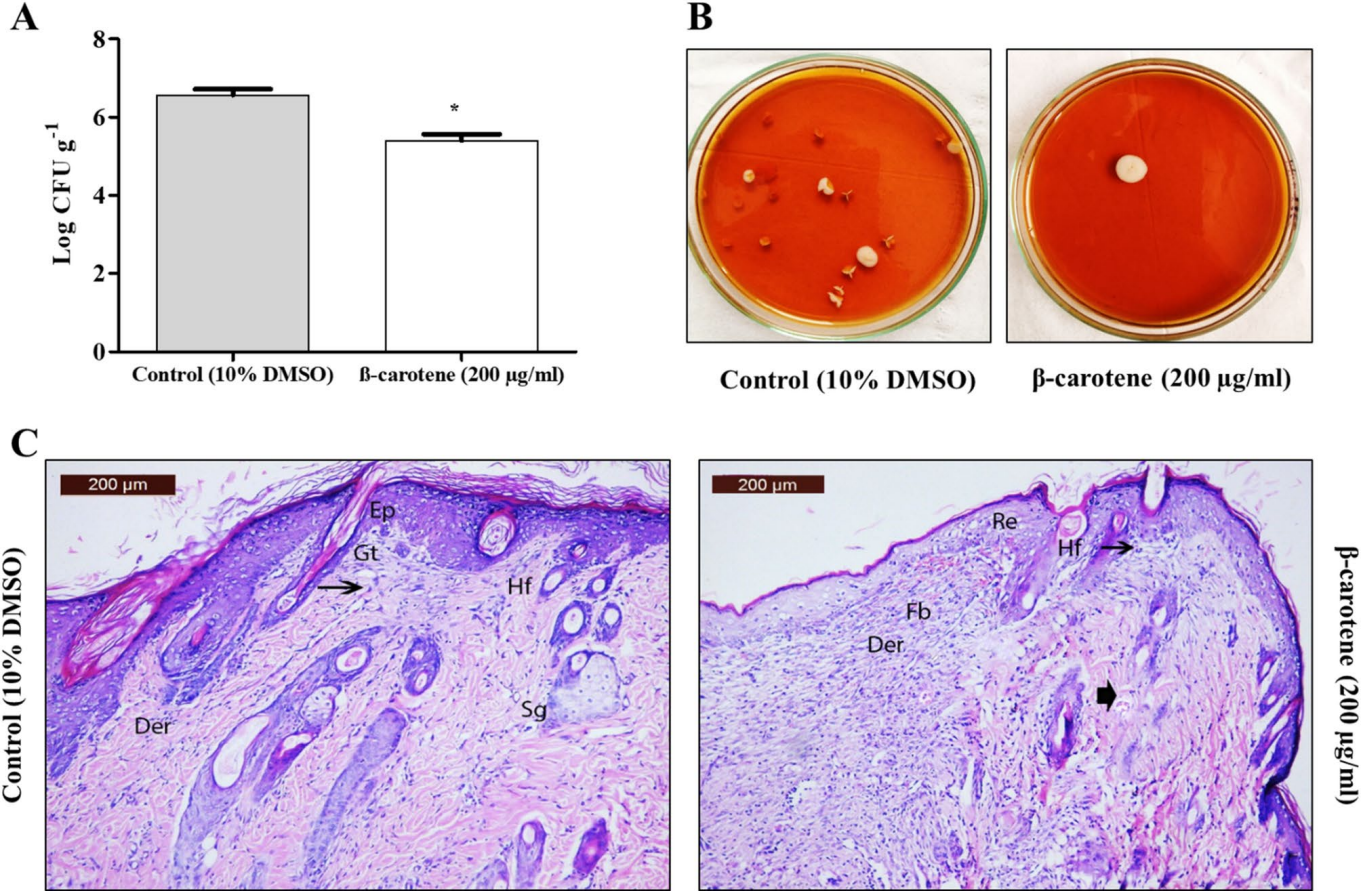
Estimation of fungal burden and histopathological examination of control (10% DMSO) group and β-carotene-treated group. Mean log wound fungal count in β-carotene-treated and untreated *C. albicans*-infected wound in rat groups (number per group = 3) (**A**). Representative SDA plates displayed the lowered fungal burden in the treated group, compared to the control group (**B**). Histological examination of wound in treated and control groups; photomicrograph of a section in rat skin from the control group showed discontinuous, thickened epidermis (Ep) with underlying granulation tissues containing sub-epidermal (Gt), necrosis of dermis (Der), loss of hair follicles and sebaceous glands (SG). Note mild re-epithelialization (Re) of the epidermal layer overlying scales with small crust containing inflammatory cells (arrow) is adhered to the epithelium at the edge of the unhealed wound surface. On the other hand, photomicrograph of a section in rat skin from treated group showed re-epithelialization (Re) in the epidermis layer, mild discontinuous epidermis (Ep), mild necrosis dermis (Der), inflammatory infiltrate cells (arrow) and an early granulation tissue composed of activated fibroblasts (Fb), with few capillaries (arrowhead) and newly formed hair follicles (HF) (**C**). The graph shows the average values of triplicate measurements and the error bars indicate standard deviations. The asterisk signifies the significance at *P* < 0.001

Before STZ therapy, rats had an average weight of 354 ± 11.4 g and a mean glucose level of 95.6 ± 9.4 mg/dl. On the wound induction day, the average weight was equal to 311.4 ± 10.8 g, and the mean glucose level was equal to 298.4 ± 43.7 mg/dl. After infection with *C. albicans* (CA 14), diabetic rats were administered 200 µg/ml of β-carotene subcutaneously in excisional wounds, and the control group received 10% DMSO. It was reported that β-carotene accelerated diabetic wound healing in the treated group compared to the control group, and the average wound diameters in the control group were 10, 8.6, 6.5, and 4.8 mm on days 0, 4, 8, and 12, respectively. In contrast, the average wound size in the β-carotene-treated group was 10, 6.2, 3.2, and 1.1 mm at days 0, 4, 8, and 12. Additionally, the control group had wound healing ratios of 14, 35, and 52% at days 4, 8, and 12, whereas the β-carotene-treated group had ratios of 38, 68, and 89% at days 4, 8, and 12 (Fig. 9). The effectiveness of β-carotene was tested by analyzing the fungal burden in diabetic rats’ infected wounds, and the results revealed that the β-carotene-treated wounds had a considerably decreased fungal burden (mean log CFU/g = 5.4) compared to untreated wounds (mean log CFU/g = 6.6) (Fig. 10A, B**)**. Furthermore, the histologic examination of tissue samples on day 12 indicated extensive damage, skin ulceration covered by acute and chronic inflammatory cells, and necrotic debris with no epithelialization in the control group. In contrast, the treated group had complete epithelization, underlying granulation tissue, and few inflammatory cells (Fig. 10C).

## Discussion

The main human pathogenic fungus is *C. albicans*. It can result in a wide range of mucosal and systemic infections and becomes a serious health issue in immunocompromised patients [39]. The prevalence of MDR *C. albicans* has exacerbated the need for novel antifungal agents [40]. Therefore, our study aims to find a new treatment option against MDR *C. albicans* infections. The antimicrobial properties of carotenoids have been documented in numerous studies [41]. In addition, these compounds have several advantages, including low toxicity, antioxidant properties, and stability in acidic environments and at neutral pH [42]. For the first time, the current study reported the antifungal activity of β-carotene against MDR *C. albicans*, as well as its antibiofilm action in eradicating pre-established biofilms of MDR *C. albicans*.

Our data revealed that β-carotene (200 µg/ml) exhibited a profound anticandidal activity against the planktonic cells of non-MDR and MDR *C. albicans*, with inhibition zones reaching up to 34 mm, and MICs ranged from 50 to 100 µg/ml. Methylene blue staining confirmed the loss of viability of fungal cells when treated with 200 µg/ml of β-carotene. On the contrary, the control (10% DMSO) showed no antibacterial activity against all tested isolates. According to our results, the positive control amphotericin-B showed inhibition zones ranging from 23 mm to 36 mm and MICs of 0.25 and 0.5 µg/ml against all tested isolates, which was 200-fold less than our tested β-carotene. In accordance with our data, Al-Taie & Al-Katib reported that β-carotene inhibited the fungal growth of *C. albicans* and *Aspergillus flavus* [43]. In addition, Madhukar reported the antifungal activity of β‐carotene pigment against some pathological fungi that attack plants, such as *F. oxysporum* NCIM 1281, *A. niger* NCIM 1025, and *P. chrysogenum* NCIM 709 [17]. The antimicrobial activity of β-carotene is related to its ability to dissolve in the lipids of the protoplasm membrane, boosting the penetration through the membrane, which either limits or kills the growth of certain pathogenic microorganisms, including bacteria and fungi [18]. In addition, according to Karpiński et al. carotenoids exert their antimicrobial action through various mechanisms, such as damaging lipopolysaccharide and nucleic acid, preventing ATP formation and oxygen uptake, disrupting the efflux pump, and increasing ROS formation inside the bacterial cell [41].

*C. albicans* frequently forms biofilms, which are a complex three-dimensional structure of microbial colonies living in an exopolysaccharide matrix [44]. These biofilms are highly resistant to antifungal agents, leading to persistent infections; therefore, it is more challenging to remove chronic diseases associated with biofilm formation [44]. For the first time, the current study demonstrated the antibiofilm impact of β-carotene against the strong biofilm-forming MDR *C. albicans*, with percentages of biofilm eradication ranging from 30.6% to 95%. In comparison, the control (10% DMSO) showed no antibiofilm eradication activity against all tested isolates, whereas the positive control manifested a strong antibiofilm activity with percentages ranging from 62% to 92%. In addition, the antibiofilm activity of β-carotene against MDR *C. albicans’* biofilms was further confirmed by simple staining, dual staining, and fluorescence staining approaches, which manifested a reduction in biofilm biomass, polysaccharide matrix, and the number of viable sessile cells within treated biofilms. As well, examination of *C. albicans* biofilm after treatment with amphotericin-B (positive control) revealed a diminished biofilm, mainly by decreasing the viability of fungal cells with negligible effect on the polysaccharide matrix. Conversely, the biofilm treated with 10% DMSO (negative control) showed a thick, developed biofilm structure with many living sessile cells surrounded by a dense layer of polysaccharide matrix. To the best of our knowledge, no study has discussed the biofilm eradication activity of β-carotene, but Majeed reported only the biofilm inhibitory activity (ranging from 5.71% to 23.84%) of carotenoid extract from *Micrococcus luteus* against *Escherichia coli*, *Bacillus subtilis*, *S. aureus*, *K. pneumoniae*, and *P. aeruginosa* at a dose of 1 mg/ml [45]. Besides, *P. aeruginosa*’s biofilm formation was reduced by 50% in the presence of 2–4 µg/ml of the *Rhodococcus* sp. SC1’s carotenoid pigment [46].

In addition, our study investigated for the first time the mechanistic pathway for β-carotene’s antibiofilm activity against MDR *C. albicans*. Our data showed that the antibiofilm action of β-carotene against MDR *C. albicans* was attributed to inhibition of the adhesive phenotype of fungal cells to initiate biofilm formation, and the percentages of inhibition ranged from 44% to 90%. As well, treatment with positive control (amphotericin-B) reduced the adhesive phenotype to a lesser degree (ranging between 20% and 37%); in contrast, the negative control (10% DMSO) manifested no effect on the adhesion capability of the tested MDR *C. albicans.* The adhesive phenotype represents the first stage in the establishment of *C. albicans* biofilm, which gives it the chance to colonize the host for initiating the biofilm cascade [47, 48]. In agreement with our study, Lee & Kim assessed the mechanism of the *Paeonia lactiflora’s* extract as an antibiofilm agent against *C. albicans*, and the effect was related to 38.4% disruption of the initial adhesion step [33]. Moreover, our data reported that β-carotene significantly reduced the expression of the *ALS3* gene by MDR *C. albicans* by 5.5-fold to 8.5-fold when compared with the control and amphotericin-B, which manifested no effect on the expression of *ALS3* gene. This gene is a member of the ALS gene family, which encodes cell-surface glycoproteins that regulate the adhesive phenotype of *C. albicans* [49, 50]. In agreement with our study, sophorolipid manifested its inhibitory effect on *C. albicans* biofilm via suppression of the *ALS3* gene expression by 8.7-fold [35]. Furthermore, our results manifested that β-carotene affected the adhesive phenotype of *C. albicans* by forming an inactive complex with Als3 protein with a binding free energy of −9.8 kcal/mol. Similarly, the antibiofilm activities of thiazolidinedione derivatives against *C. albicans* biofilm were attributed to obstructing the adhesive role of Als3 protein via complex formation with binding free energies ranging from − 9.16 to −10.81 kcal/mol [51]. Also, El-Baz et al. examined the antibiofilm activity of essential oils, such as clove, cinnamon, jasmine, and rosemary, against *C. albicans* biofilm and it was found that these oils bound to the Als3 protein and blocked its function, with binding free energies of −9.38, −8.03, −8.19, −8.81, and − 7.01 kcal/mol, respectively [37].

The existing study also reported that the biofilm eradication activity of β-carotene was dependent on the complete inhibition of yeast-to-hyphae transition in MDR *C. albicans*. In contrast, positive control (amphotericin-B) and negative control (10% DMSO) revealed no negative effect of hyphae formation. Hyphae are a crucial structural element that contributes to the overall architectural integrity of *C. albicans* biofilms [9]. In accordance with our data, 19, 20-epoxycytochalasin Q and 3-hydroxy coumarin exhibited their antibiofilm action against *C. albicans* by inhibiting hyphal formation [52, 53]. Besides, our study screened the effect of β-carotene on the viability of sessile cells via CLSM, CFU assay, and methylene blue staining. The resultant data displayed that β-carotene activated cell death of sessile cells in *C. albicans* biofilms, with a percentage of reduction in viability reaching up to 80%. Also, treatment with amphotericin-B also showed a reduction in viability with a bit higher degree (up to 83.5%). On the other hand, the 10% DMSO displayed no impact on the viability of sessile cells. Furthermore, our tested β-carotene inhibited the production of EPS, and the percentages of inhibition ranged from 55.6% to 95.6%. In contrast, the negative control (10% DMSO) and positive control (amphotericin-B) exhibited no effect on EPS production by the tested isolates. According to previous studies, the EPS production plays a vital role in the maintenance and assembly of biofilm [11, 54]; therefore, lowering EPS critically disrupted biofilms of *C. albicans*.

One of the biggest health issues for people with type 2 diabetes is fungal infections [55]. According to a study by Fata et al. fungal infections are seen in over 20% of diabetic foot cases and are highly resistant to antifungal treatments, resulting in skin lesions with a bad prognosis [56]. Mycotic diabetic foot is most frequently caused by different species of *Candida*, particularly *C. albicans* [46]. Subsequently, the effect of β-carotene on MDR *C. albicans*-infected diabetic wounds was investigated for the first time in the current study. From wound measurements, it was observed that β-carotene ameliorated diabetic wound healing in the treated rat group, and the healing capacity was up to 89% at day 12. As well, the fungal burden severely declined in the treated group (mean log CFU/g = 5.4). On the contrary, the healing of diabetic wounds in the control rat group was delayed (52% at day 12), as well as the control group demonstrated a higher fungal burden (mean log CFU/g = 6.6). Furthermore, histological examination of the treated group manifested regeneration of skin with few capillaries and newly formed hair follicles. On the other hand, the skin lesions of the control group displayed extensive damage, skin ulceration, and necrotic debris with no epithelialization. In agreement with our results, the topical application of carotenoid (astaxanthin) accelerated full-thickness wound healing in mice [57], and according to Chong et al. carotenoids can promote wound healing by various mechanisms, including enhancing cell proliferation, reducing inflammation, and scavenging free radicals [58]. When it comes to its toxicity, β-carotene’s toxicity is generally low [59], and it was the first approved food additive in 1995 [60]. Additionally, β-carotene’s safety was examined, and no adverse effects were observed following clinical pathology and histological evaluations of rats given a dose of 500 mg/kg of β-carotene [61, 62].

In summary, our study reported that β-carotene showed a potent antifungal activity against all tested isolates, with inhibition zones reaching 34 mm. In addition, β-carotene eradicated MDR *C. albicans’* biofilms by inhibiting the adhesive phenotype, yeast-to-hyphae transition, sessile cell viability, and EPS production. Moreover, the diabetic wound model confirmed the anticandidal activity of β-carotene. The clinical application of β-carotene in treating diabetic wounds infected by MDR *C. albicans* may be promising in the future.

## Supplementary Information

Supplementary Material 1.

## Data Availability

All data generated or analyzed during this study are included in this published article.
