# Supplementary material for: The dual antifungal and antibiofilm activities of β-carotene against multidrug-resistant Candida albicans induce wound healing in a diabetic rat model: an in vitro and in vivo study
Source: BMC Microbiol. 2025 Nov 15;25:747. doi: 10.1186/s12866-025-04447-w (PMC12619372; doi:10.1186/s12866-025-04447-w)
Supplement: Supplementary file 1 — Supplementary Material 1. [file 12866_2025_4447_MOESM1_ESM.docx]

**Table S1** Primers of the *ACT1* and *ALS3* genes in qRT-PCR.

| Genes | Primer sequence (5′-3′) |
| --- | --- |
| *ACT1* | F: GGTTTGGAAGCTGCTGGTATTGACC |
|  | R: ACGTTCAGCAATACCTGGGAACATG |
| *ALS3* | F: CAACATCAACCAACCAATCTC |
|  | R: TGAATAACAGAACCAGATCCG |

**
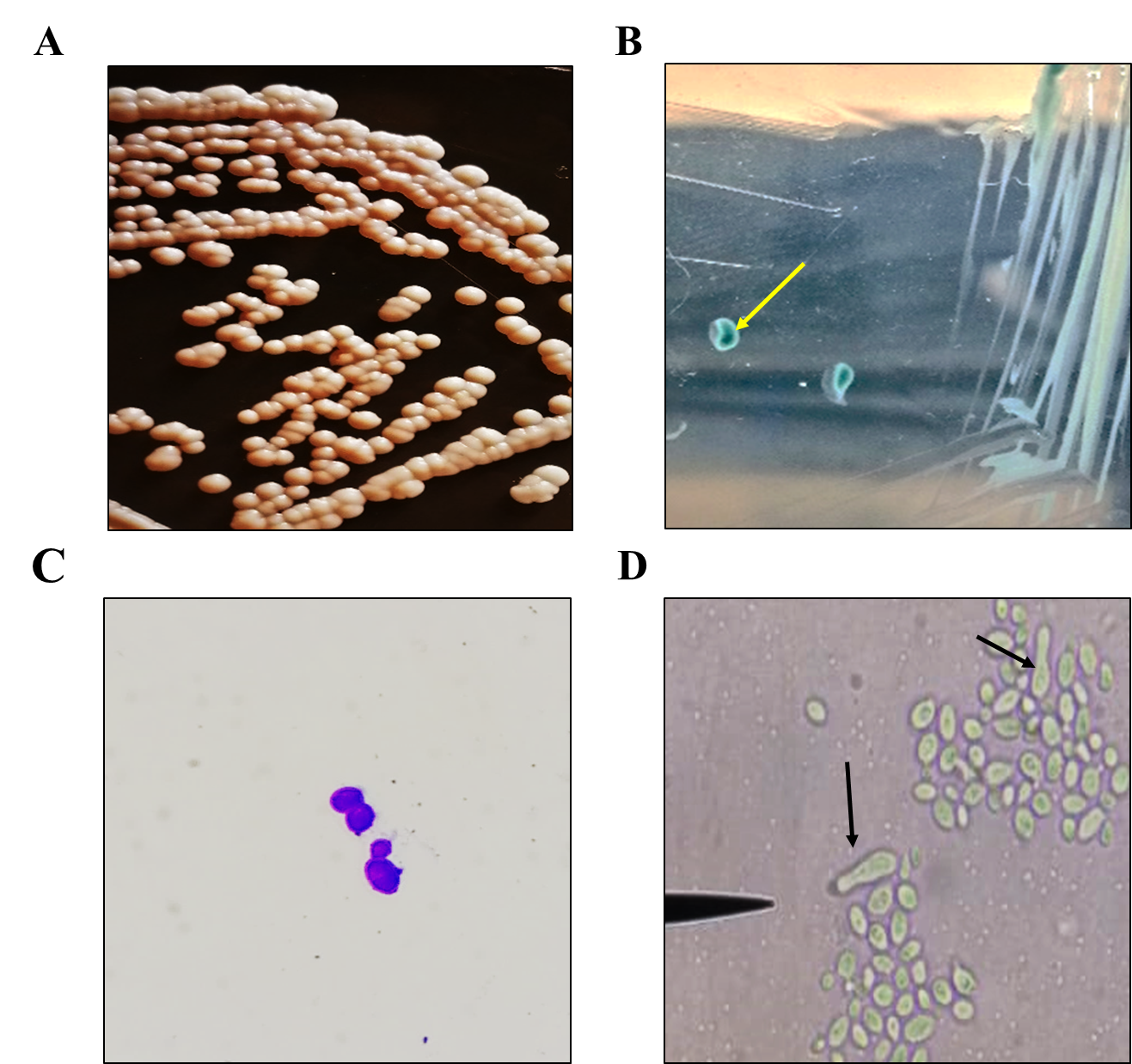
**

**Fig. S1** Identification of *C. albicans* isolates. Creamy, smooth, and pasty convex colonies of *C. albicans* on SDA (**A**). Green colonies (yellow arrow) of *C. albicans* on CHROMagar™ Candida medium (**B**). Gram staining of *C. albicans* showing Gram-positive fungus (**C**). Germ tube (black arrow) of *C. albicans* under light microscope (100x) (**D**).


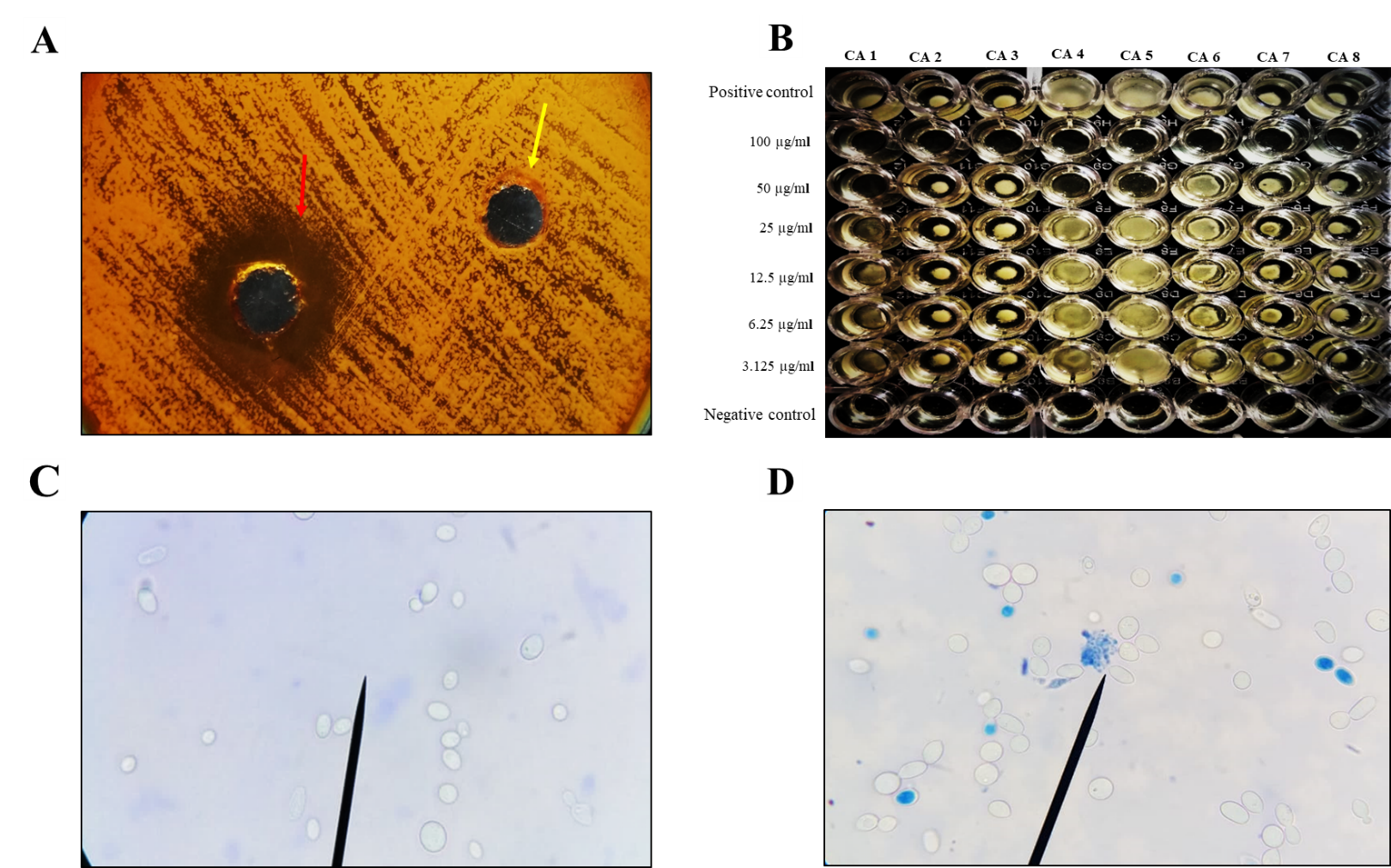


**Fig. S2** Antifungal activity of β-carotene (200 µg/ml) against *C. albicans*. Well diffusion assay showed the resultant inhibition zone (red arrow), where 10% DMSO acts as a negative control, showed no inhibition zone (yellow arrow) (**A**). A microtiter plate visualized against a black background to determine the MIC of β-carotene against *C. albicans* using the microdilution method (**B**). Unstained live cells of untreated *C. albicans* (**C**). Methylene Blue-stained dead cells of *C. albicans* after treatment with β-carotene (200 µg/ml) for 3 hr (**D**).

**
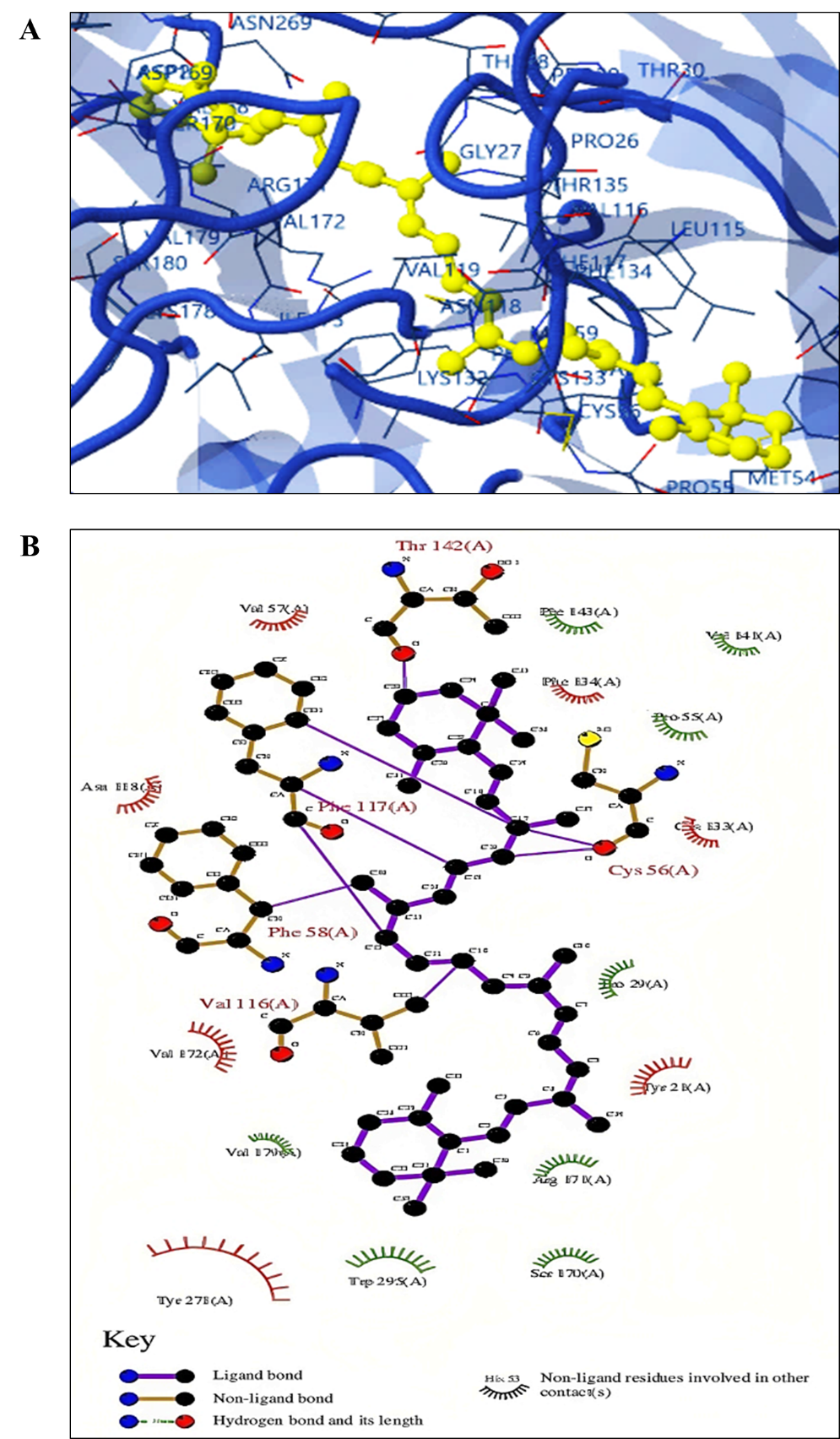
**

**Fig. S3** Docking of β-carotene with Als3 protein. The 3D structure revealed the fitting of β-carotene (yellow color) in the active site of the Als3 protein (**A**). The 2D structure showed the interacting amino acids from Als3 protein with β-carotene (**B**).
